# Nitric oxide required for transition to slower hepatic protein synthesis rates during long-term caloric restriction

**DOI:** 10.1172/JCI189798

**Published:** 2026-01-02

**Authors:** Hector H. Palacios, Edward Cao, Adelaide Cahill, Hussein Mohamad, Marc K. Hellerstein

**Affiliations:** 1 Center for Human Nutrition, Washington University in St. Louis, St. Louis, Missouri, USA.; 2 Nutritional Science and Toxicology Department, University of California — Berkeley, Berkeley, California, USA.

**Keywords:** Aging, Hepatology, Metabolism, Nitric oxide, Proteomics

## Abstract

Calorie restriction (CR) extends maximal lifespan and maintains cellular homeostasis in various animal models. We have previously shown that CR induces a global reduction of protein fractional synthesis rates (FSRs) across the hepatic proteome in mice, but the timing and regulatory mechanisms remain unclear. Nitric oxide (NO), a bioactive molecule upregulated during CR, is a potential regulator of protein synthesis. To explore the role of NO in hepatic proteome fluxes during CR, we used in vivo deuterium labeling from heavy water and liquid chromatography/mass spectrometry–based (LC/MS-based) flux proteomics in WT and NO-deficient (NO^–^) mice. We observed a transition to reduced global protein FSRs that occurred rapidly between days 25 and 30 of CR. NO deficiency, whether genetic or pharmacological, disrupted the slowing of proteome-wide fluxes and the beneficial effects on body composition and physiology. Administering the NO donor molsidomine restored the reduction in hepatic FSRs in NO^–^ mice. Furthermore, inhibiting NO pharmacologically, whether starting on day 1, day 14, or day 24 of CR, mitigated the reduction in hepatic protein FSRs at day 32, highlighting NO’s critical role during the transition period. These results underscore the importance of NO in CR-induced changes in proteostasis and suggest NO as a potential CR-mimetic target, while offering a specific time window for identifying other signals and testing therapeutic interventions.

## Abstract 2

Nitric oxide is essential for caloric restriction-induced reduction in liver protein synthesis, occurring as a rapid transition event between weeks 4-5.

## Graphical abstract

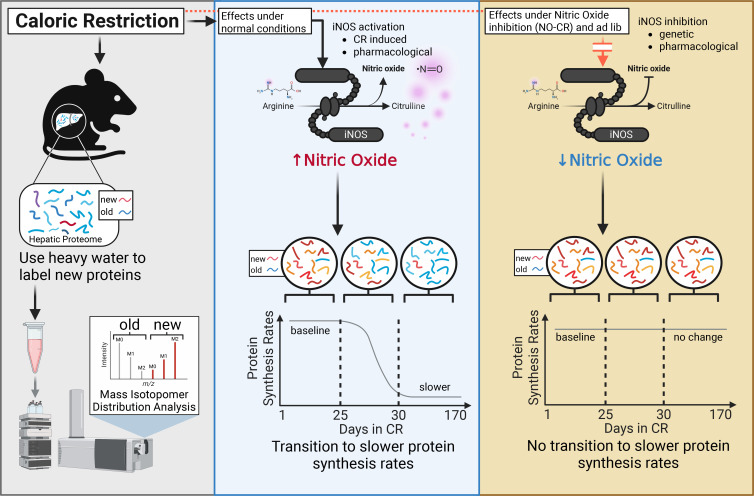

## Introduction

Calorie restriction (CR) is widely acknowledged as an effective intervention for extending healthspan and lifespan in animal models (1–4). We (2, 5, 6) and others (7–9) have reported that CR substantially reduces proteome-wide hepatic protein synthesis and breakdown rates in mice, resulting in longer protein half-lives and reduced absolute protein synthesis. This proteostatic phenotype has also been confirmed in CR-mimetic mouse models with lifespan extension, such as mice administered rapamycin and Snell-Dwarf mice (5). Studies in lower organisms (e.g., *Caenorhabditis* *elegans*) also reveal evidence for general slowing of protein translation efficiency during CR (10–14). However, the underlying regulatory mechanisms and signals remain unknown. Therefore, it is important to understand how global protein turnover is regulated and to examine the detailed time course involved in the transition to slower protein synthesis rates during CR (2, 6, 15, 16).

Aging impairs cellular function, in part through increased oxidative stress and diminished nitric oxide (NO) bioavailability (17). Under physiological conditions, NO plays a critical role in a wide range of biological functions, including signaling, homeostasis maintenance, and immune response (18–20). The reduction of NO during aging may partially explain the increase in inducible NO synthase (iNOS) expression as a compensatory mechanism (21). In contrast, during CR, iNOS expression decreases in association with reduced oxidative stress and improved metabolic function (17, 21, 22) and may mitigate cellular damage by enhancing NO production (17–19, 22), reducing superoxide production, and modulating nutrient-sensing pathways like mTOR (23).

NO potentially represents a strategic target for CR interventions aimed at improving health and combating the deleterious effects of aging (24). Of the diverse pathways known to change under CR, many coalesce at the point of protein translation. NO, through signal transduction pathways, can mediate the inhibition of protein synthesis through dephosphorylation of the translation initiation factor 4E-BP1 (25). In addition, NO-mediated regulation of gene expression involves the activation of multiple kinases that phosphorylate the translation initiation factor eIF2 (15, 26), leading to its inactivation and a reduction of translation initiation and repression of global protein synthesis (26).

Taken together, these factors motivated the current studies aimed at investigating, in fine detail, the timing of CR-induced slowing of hepatic protein turnover rates and the role of NO in this process. We examined liver tissue from mice on a CR diet, which involved a 30% reduction in food intake compared with the ad libitum (ad-lib) control (Con) group over a period of 9 days up to 170 days. The CR mice were provided food daily at 1200 hours, ensuring a regular feeding schedule. We used in vivo heavy water labeling and liquid chromatography tandem mass spectrometry–based (LC-MS/MS-based) flux proteomics based on mass isotopomer distribution analysis (MIDA) (2, 5, 27, 28) to measure protein fractional synthesis rates (FSRs) in proteins across the liver proteome. Our hypotheses were: (a) that the time course of CR-induced slowing of global hepatic protein synthesis rates will reveal clues to the underlying signals involved; (b) that alterations in hepatic protein synthesis rates will not parallel changes in hepatic gene expression; (c) that there will be a marked role of iNOS in the effects of CR on hepatic proteome fluxes and on the physiological and biological consequences of CR; and (d) that deficiency of NO production (NO^–^), either genetically or pharmacologically, will also lead to unfavorable physiologic outcomes, in addition to inhibiting the effects of CR on hepatic proteome fluxes, and that these effects will be reversed by pharmacologic replacement of NO.

## Results

### CR time course and in vivo metabolic labeling procedures for proteome-wide synthesis rates.

**Figure 1:**
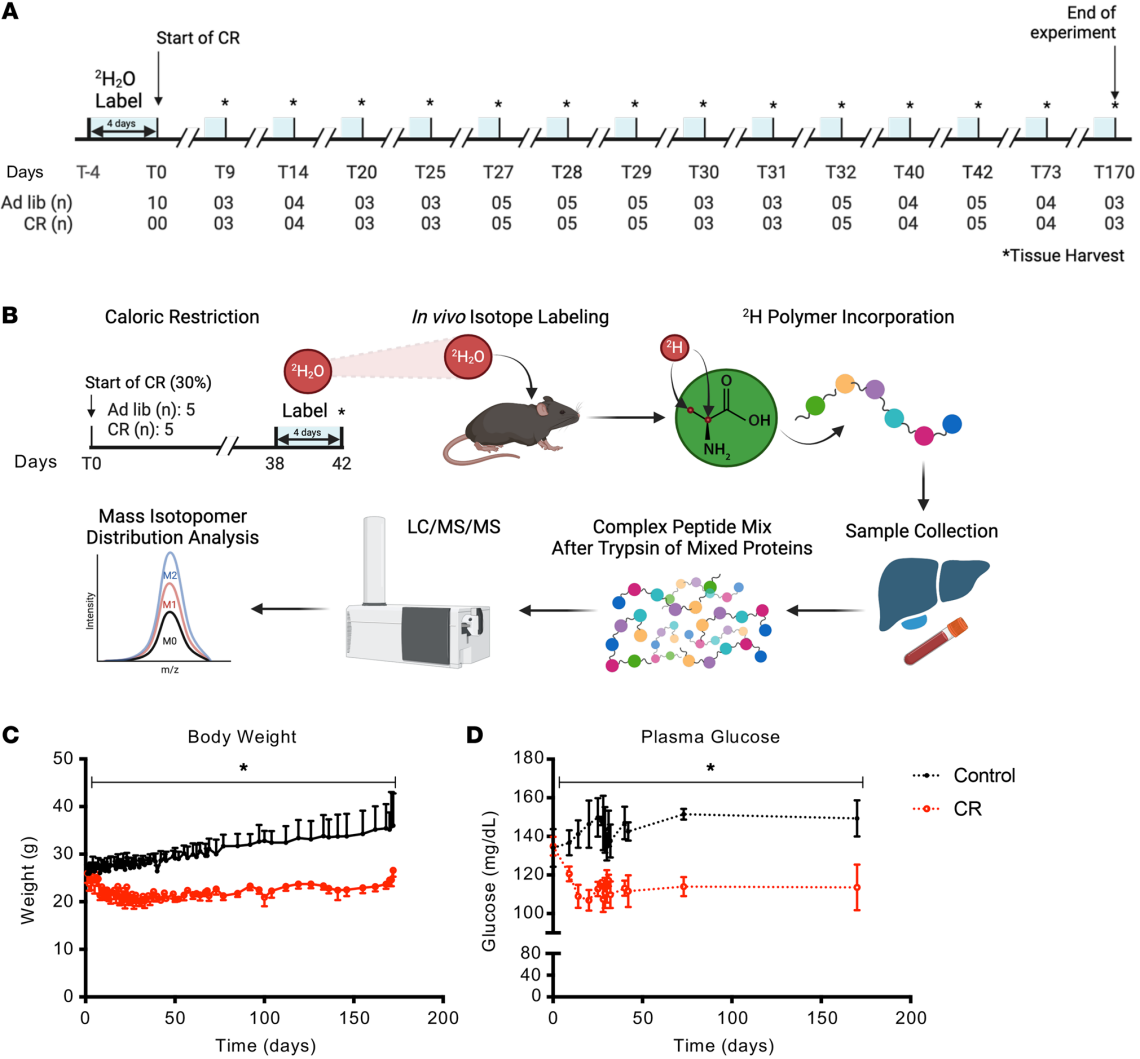
CR time-course experimental design for hepatic proteome–wide fluxes. (**A**) Experimental time points. Male C57Bl/6J mice, starting at 6 weeks of age, underwent CR (30% fewer calories than mice in the Con group) for up to 170 days, with CR feeding scheduled at 1200 hours to ensure a regular feeding schedule. Starting 4 days before sacrifice, mice were labeled with a ^2^H_2_O by i.p. bolus, and then body water enrichment was maintained with 8% ^2^H_2_O in drinking water. (**B**) Experimental approach for measuring in vivo protein fluxes (FSRs) across the global proteome. (**C**) Body weights. Mice (*n* = 120) were single-caged and split into ad-lib (Con) and CR groups. Mice were weighed at regular intervals throughout the experimental period. (**D**) Body weight and plasma glucose levels in mice fasted for 5–6 hours are shown for the Con and CR groups. Data indicate the mean ± SD (*n* = 3–5 for all experiments).*P < 0.05 versus Con, determined by repeated-measures ANOVA.

To investigate the effect of CR on protein synthesis rates across the global hepatic proteome and gain a deeper understanding of the time course and underlying signaling mechanisms, we conducted an intensive time-course study using C57BL/6J mice (*n* = 120). Starting from 6 weeks of age and after an acclimatization period, the mice were individually housed and subjected to CR, in which they consumed 30% less food compared with the Con mice. We included the following time points for in vivo labeling studies: 0 days (baseline Con), 9, 14, 20, 25, 27, 28, 29, 30, 31, 32, 40, 42, 73, and 170 days (Figure 1A). Age-matched Con mice, which were not subjected to CR, were used to establish 0 days as the baseline for protein turnover rates and to assess the variability of our flux proteomics measurements under ad libitum feeding conditions. The day 0 Con mice were labeled for 4 days prior to the assessment of hepatic protein kinetics. The coefficient of variation (CV) in these baseline samples served as a reference point for statistical analyses of all subsequent time points. We then selected time points aimed to capture the early, transitional, and long-term effects of CR on hepatic proteome turnover (Figure 1A).

We used stable isotope labeling and MS/MS analysis with the MIDA approach (2, 5, 27, 29) to measure hepatic protein turnover rates (FSRs) as the primary outcome of this study. This approach involves i.p. injection of 99.8% ^2^H_2_O to increase the ^2^H enrichment of body water in mice to approximately 5%. Subsequently, we provided 8% ^2^H_2_O in the drinking water to maintain the body water ^2^H-enrichment constant at approximately 5% during the labeling period (2, 5, 30). Hepatic proteins were extracted and subjected to trypsin digestion (2, 5, 27). The resulting peptides were analyzed using LC-MS/MS, allowing for identification and kinetics analysis of the parent proteins (Figure 1B).

Body weight is an important assessment of CR that changes in less than 10 days following intervention (31, 32). To closely monitor changes throughout the study, we conducted regular body weight measurements. We observed a significant decrease in body weight (*P* < 0.05) within the first week of initiating the experiments (Figure 1C). The average body weight of the mice in the CR group was 26.0 ± 2.0 g at 6–7 weeks of age (start of CR) and 26.6 ± 1.4 g at the final time point (31 weeks of age) (Figure 1C). Weight regain and stabilization were consistent with previous observations that C57BL/6 mice regain weight over time during prolonged CR (33, 34).

Short-term CR can improve glucose homeostasis in male mice (35), so we measured plasma glucose levels (Figure 1D) after a 6-hour fast. This measurement revealed a significant effect of CR in lowering fasting plasma glucose concentrations (*P* < 0.05).

### Time course of hepatic proteome-wide protein synthesis rates.

**Figure 2:**
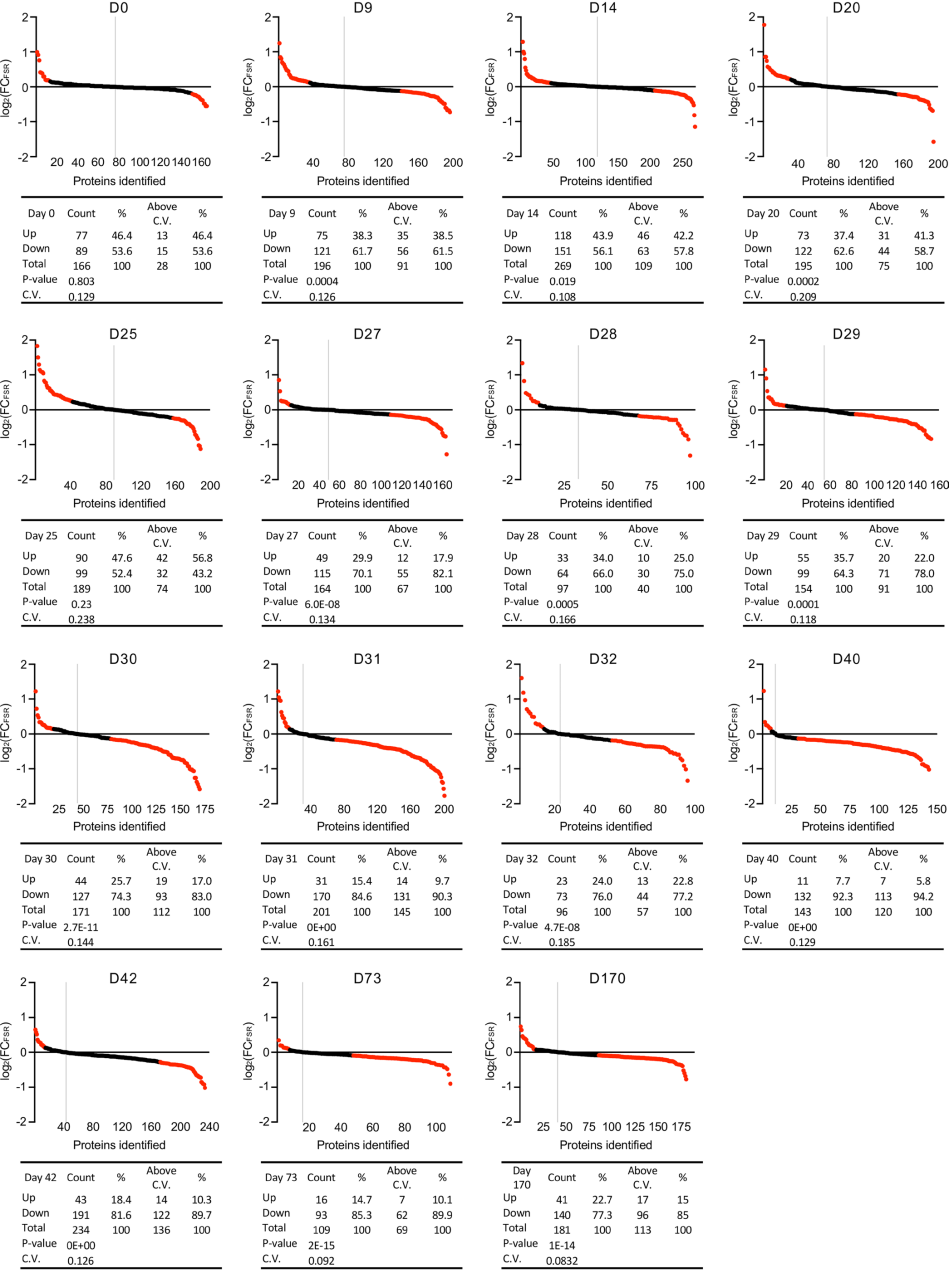
Time course to CR threshold event transition to slower hepatic protein FSRs. Time course of reduction in proteome-wide hepatic protein FSRs during long-term CR. Data represent experiments in which C57Bl/6J male mice underwent CR for different lengths of time, with heavy water labeling for 4 days prior to each termination time point shown. Livers were collected and proteome flux rates were analyzed by LC-MS/MS. Protein FSRs (turnover rates) were calculated by MIDA, as described in Methods. The data show the FSR as log_2_ values of the FC between the CR and Con groups. Dots represent individual proteins identified by LC-MS/MS, sorted from highest to lowest value. Above the “0” line are the proteins with a higher FSR under CR, while the dose below the “0” line represents the proteins whose FSR was lower under CR. To establish a cutoff threshold value, the CV was averaged, and this average value was used as the cutoff — i.e., a percentage change greater than that of the CV average was considered to be above the cutoff and is marked in red. Vertical gray lines show the dividing line separating faster (left) and slower (right) FSRs. The tables reflect the graphed data and represent the number and percentage of proteins upregulated (higher FSR) or downregulated (lower FSR) in CR compared with the Con. *P* values show the binomial distribution significance, determined using a 2-tailed binomial distribution. These results are consistent with a modest reduction before day 25 in hepatic FSRs (50%–60% of proteins with lower FSRs than Con) that rarely passes the CV cutoff until after days 25–30. After this transition, there was a global reduction, with 75%–90% of proteins having lower FSRs that exceeded the cutoff compared with the Con. D0, day 0.

From the comprehensive list of detected proteins that passed analytic criteria (2, 27, 36), we paired all proteins in each group at each time point and evaluated the log_2_ fold change (FC) of each protein’s FSR (CR over Con) to assess the effects of CR on hepatic protein FSRs (Figure 2). Proteins that had a higher value in the CR group than the Con group were classified as “Up” in response to CR. Conversely, if the FSR value was lower in the CR group, the protein’s FSR was classified as “Down.” Each time-course experiment was sorted from the highest log_2_(FC_FSR_) value to the lowest. To evaluate significance, we performed a binomial statistical test (i.e., the probability that *x* number of coin tosses would result in the distribution of Up vs. Down FSRs), presenting the results as 2-tailed *P* values, which are quantified in Figure 2. Additionally, we analyzed proteins represented across all time points (Supplemental Figure 3). This analysis indicates no significant changes in hepatic proteome kinetics prior to 25 days of CR. However, a transitional period is observed between 25 and 30 days, followed by a marked global reduction in FSR values after 30 days of CR.

To establish a cutoff for statistical stringency in binomial comparisons, we calculated the CV for each protein for the Con and CR groups. We then used the average of all the CVs as a proxy for variability within the experiments (37–39). Any change in value greater than the average CV was considered above the cutoff and is highlighted in red in Figure 2. This approach provided us a comprehensive method to determine the effect of CR on protein turnover rates and to identify proteins that exhibited substantial changes in response to interventions.

### Observing a transition period or threshold event in hepatic proteome FSRs during CR.

**Figure 3:**
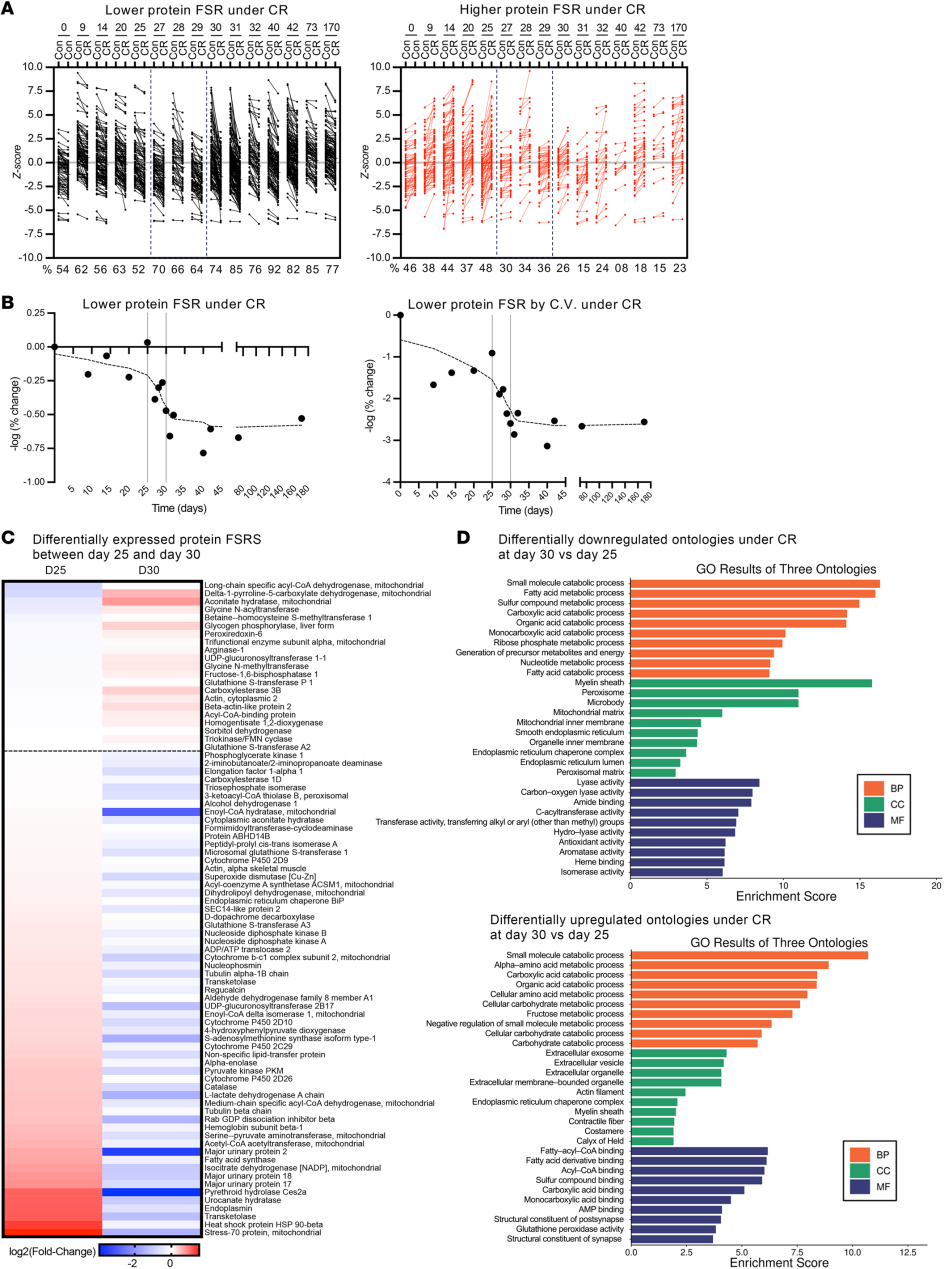
Identifying the threshold event time point at which CR induces a dramatic slowdown of protein turnover rates. (**A**) Each dot represents the FSR as a *z* score for every identified protein. In black (left) are the proteins whose FSR was lower after CR compared with the Con. In red (right) are the proteins whose FSR were higher after CR compared with the Con. The bottom of the graph shows the percentage of total proteins at each time point. Note that the percentage of proteins with a higher FSR is smaller in the right graph, especially after day 30. (**B**) The percentage values of the proteins that had a lower FSR under CR are shown as the –log_2_ FC relative to day 0. The top panel shows the –log_2_ (FC) without adjusting for the CV. The bottom panel shows the –log_2_ (FC) relative to the percentages adjusted for CV. (**C**) Heatmap of the log_2_ FC between CR and Con FSRs. Heatmap represents the proteins whose FSR changed between days 25 and 30, with the differentially expressed proteins between the 2 time points shown. (**D**) GO framework by aspect was used to analyze pathway enrichment analysis. BP, biological process; CC, cellular component; MF, molecular function.

To facilitate a comprehensive understanding of the proteome-wide FSR time-course changes (Figure 2), we summarized the data using a standard score (*z* score), where each FSR value was positioned relative to the mean of the group. Through this standardization, each identified protein FSR allowed for easier comparisons between Con and CR across the different time points (Figure 3A). The number of individual proteins with either a lower FSR after CR intervention (Figure 3A, depicted in black on the left) or a higher FSR (Figure 3A, depicted in red on the right) are shown at each time point. The percentages shown indicate the proportion of proteins that exhibited either lower or higher FSR values under CR. The distribution of these changes was day 0: 54% down, 46% up; day 9: 62% down, 38% up; day 14: 56% down, 44% up; day 20: 63% down, 37% up; day 25: 52% down, 48% up; day 27: 70% down, 30% up; day 28: 66% down, 34% up; day 29: 64% down, 36% up; day 30: 74% down, 26% up; day 31: 85% down, 15% up; day 32: 76% down, 24% up; day 40: 92% down, 8% up; day 42: 82% down, 18% up; day 73: 85% down, 15% up; and day 170: 77% down, 23% up.

Using these percentage values after logarithmic transformation (–log_2_), we examined the distribution of proteins with decreased FSRs under CR over time (Figure 3B, top panel). We also evaluated changes in variability using CV values, which showed a 3-fold greater degree of change (Figure 3B, bottom panel).

These analyses revealed a clear inflection pattern. During the first 25 days of CR, FSR values exhibited relatively minor changes, fluctuating between 52% and 63% of proteins showing modest downregulation (FSR <25% change). After day 25, a transition to more prevalent lower FSR values emerged, with 74%–85% of proteins affected by days 30–32, with an almost vertical slope and a doubling of the degree of change observed compared with before day 25 (Figure 3B). This period around 4–5 weeks after the start of CR represents a transitional event that suggests a threshold effect. This high prevalence of lower FSR values across the proteome was maintained for the remainder of the time course. Notably, when considering the values that surpassed the CV cutoff, the statistical differences became more pronounced (Figure 3B).

The identification of a transition event between days 25 and 30 of CR allowed us to generate a heatmap using the log_2_ FC between CR and Con FSRs depicting uniquely up- or downregulated fluxes that separate this important period (Figure 3C). Specific proteins that were differentially expressed (opposites between day 25 and day 30 by their FC) by being “uniquely” upregulated or downregulated under CR at day 30 compared with day 25 were identified (Figure 3C).

To further characterize the differences in protein expression between day 25 and day 30 of CR, we analyzed protein FSRs using Gene Ontology (GO) for proteins uniquely decreased or increased at day 30. As expected, pathways related to body composition and weight loss, such as fatty acid metabolic processes, were downregulated at day 30. Interestingly, differentially upregulated GO Biological Processes at day 30, not observed on day 25, included amino acid metabolic processes (Figure 3D).

Label-free quantitative proteomics (40–42), followed by within-proteome absolute synthesis rates (WPASRs) (5) at both day 25 and day 30, using a normalized signal intensity quotient (Q) was performed to validate our observations. Principal component analysis (PCA) (Supplemental Figure 4A; supplemental material available online with this article; https://doi.org/10.1172/JCI189798DS1) shows that CR at day 30 was the principal distinct component. Sample normalization of the WPASR (adjusted to the median for systemic differences among samples, followed by log_10_ transformation on individual values and data scaled to the mean center) are represented on the heatmap in Supplemental Figure 4B and show marginal changes at day 25 of CR, where only 56.7% of WPASRs were decreased under CR compared with its time-matched Con. Meanwhile, at day 30 of CR, 71.4% of WPASRs were decreased.

### Comparing hepatic protein FSRs with gene expression.

**Figure 4:**
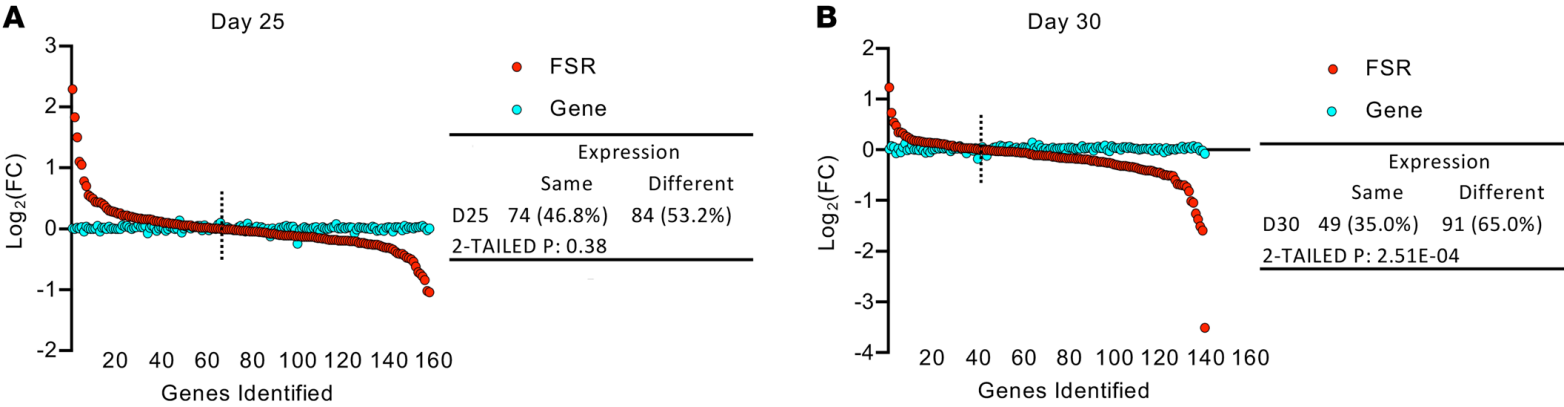
Comparison of the hepatic protein FSR with gene expression. Protein FSRs and gene expression (see Supplemental Figure 1 for overview) were matched and compared for day 25 (**A**) and day 30 (**B**). Only those targets identified in both the flux proteomics and RNA-Seq dataset were included. Proteins are shown as sorted from highest to lowest by FC of the protein FSR. Table represents the number of genes and protein FSRs with either the same or different direction of expression. Tables show statistics as determined by binomial distribution 2-tailed *P* values. Vertical dotted line separates faster (left) and slower (right) FSRs. These findings reveal that changes in protein FSRs during CR, including changes between days 25 and 30 of CR, were not reflected in mRNA measurements.

To gain a comprehensive understanding of gene expression during CR over the course of the transitional period (days 2–30), we performed RNA-Seq on hepatic mRNA for these 2 time points (Figure 4). Filtering out low-quality reads yielded 15,082 genes. Matching and comparing the expression of protein FSRs and gene expression (see Supplemental Figure 1 for overview) were conducted for day 25 (Figure 4A) and day 30 (Figure 4B), with a focus on targets identified in both the proteomics and RNA-Seq datasets. The proteins were then sorted from highest to lowest on the basis of the protein FSRs. The resulting tables in Figure 4, A and B, present the number of genes and protein FSRs with concordant or discordant expression patterns. Statistical analysis using binomial distribution 2-tailed *P* values was applied to generate the tables in Figure 4, A and B. Additionally, a vertical dotted line is shown to distinguish faster (left) from slower (right) FSR values. This analysis reveals that systematic changes in protein FSRs during CR, including those occurring between days 25 and 30, are not manifested in mRNA measurements.

A biologic “lag” between gene transcription and protein translation (43) may explain why fluctuations in mRNA levels did not correspond to changes in protein levels. Our labeling approach analyzed protein turnover rates over a period of 4 days, however, which is much longer than the usual time for the message to be translated for protein synthesis (43, 44). These findings suggest that posttranscriptional regulatory mechanisms are at work here.

### Exploring the role of NO in hepatic proteome fluxes.

**Figure 5:**
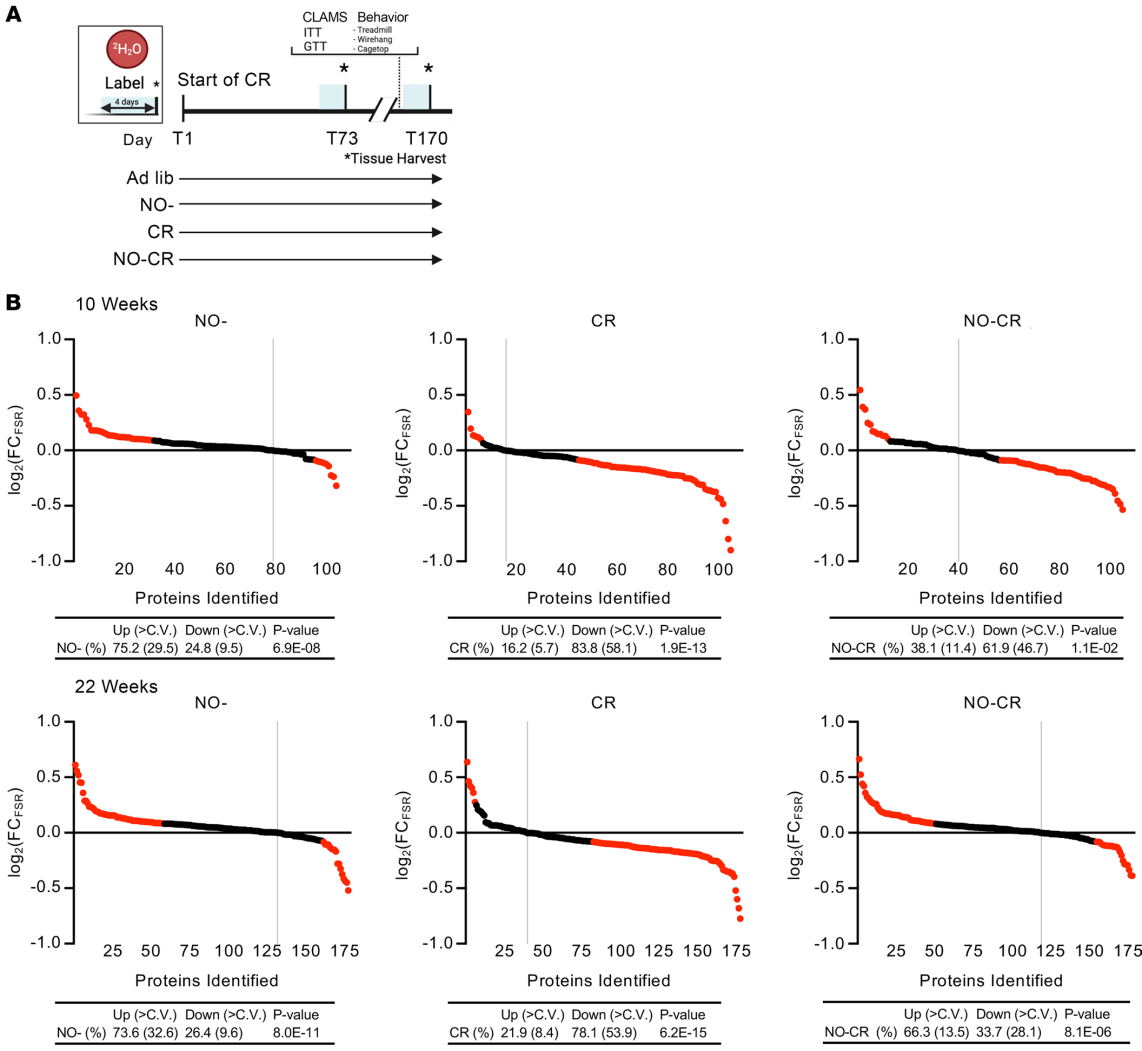
Effects of iNOS KO and CR on hepatic proteome FSRs. (**A**) Mouse experimental workflow. Mice underwent CR plus ^2^H_2_O labeling for different lengths of time. iNOS-KO mice (NO^–^) under CR at 2 time points (10 or 22 weeks of the study. Before sacrifice, mice were labeled with ^2^H_2_O for 4 days. NO^–^ mice (*n* = 20) and Con C57Bl6/J mice (*n* = 24), starting at 6–7 weeks of age, were randomly divided into 4 groups: Con ad-lib (*n* = 12); NO^–^ ad-lib (*n* = 10); CR (*n* = 12); and NO-CR (*n* = 10). (**B**) FSR values were obtained for the liver proteome at 10 or 22 weeks of intervention for Con, iNOS-KO (NO^–^), and CR iNOS-KO (NO-CR) mice. Dots along the line represent identified proteins sorted from the highest FSR to the lowest FSR for the group by the log_2_ FC compared with the ad-lib Con group. Comparisons were done for all groups. Tables represent the binomial distribution of proteins with an increased FSR (Up), or decreased (Down). In red are the proteins whose change was greater than the CV.

We then asked whether there is a role for NO in the regulation of the hepatic proteostatic response to CR. To establish the broad time course of changes, we first placed iNOS-KO mice (B6.129P2-^Nos2tm1Lau^/J) (45–47) (NO^–^ mice) under 30% CR for either 10 weeks (time [T]: T73) or 22 weeks (T170). Prior to tissue collection, the mice were labeled with ^2^H_2_O for a period of 4 days. Both NO^–^ mice (*n* = 20) and Con C57Bl6/J mice (*n* = 24) were started on the study protocol at 6 weeks of age and were randomly divided into 4 groups: Con (*n* = 12), NO^–^ (*n* = 10), CR (*n* = 12), and NO-CR (*n* = 10) (Figure 5A).

To compare flux differences, we calculated the log_2_ FC (log_2_[FC_FSR_]) values for each protein compared with the ad-lib Con, as described above. This allowed us to determine the distribution of proteins with higher (Up) or lower (Down) FSR values on a global scale. Statistical analysis was performed using binomial distribution analysis with a 2-tailed *P* value. Additionally, to enhance the rigor of our analysis, we considered proteins with changes that exceeded their CV thresholds.

We first saw, after 10 weeks (Figure 5B, top panel), that CR alone led to a marked slowdown in hepatic protein FSRs, with approximately 83.8% of proteins displaying a lower FSR compared with the Con group at 10 weeks. When considering values above the CV threshold, 58.1% had a lower FSR, while only 5.7% had a higher FSR compared with the Con group. Interestingly, these effects were diminished in the NO-CR group, with approximately 61.9% of proteins showing a lower FSR. When considering the CV threshold, approximately 46.7% of proteins in the NO-CR group exhibited a lower FSR. To test the significance of changes in protein downregulation between the CR and NO-CR groups at 10 weeks (quantified in Table 1), we used the Fisher’s exact test, which showed a significantly decreased effect between these 2 groups (*P* = 0.008).

In contrast, the NO^–^ group had distinctly higher hepatic protein FSRs, with 75.2% of proteins exhibiting a higher turnover rate compared with the Con group. Among these changes, approximately 29.5% of proteins had a significantly higher FSR above the CV, while only 9.5% had a slower FSR compared with the Con group.

At 22 weeks (Figure 5B. bottom panel), the NO^–^ group had higher FSRs (73.6%) in hepatic proteins compared with the Con group. Among proteins above the CV, 32.6% had a higher FSR, while 9.6% had a lower FSR than did the Con. CR alone decreased the hepatic proteome FSRs by 78.1%, with 53.9% of proteins above the CV being downregulated, while only 8.4% were upregulated. Notably, the NO-CR group showed significantly higher FSR values than did the Con group (*P* = 8.1^–6^), with 66.3% of proteins being upregulated. However, when considering values above the CV, only 13.5% had a higher FSR, while 28.1% had a decrease in the FSR. We performed Fisher’s exact test to compare protein FSR downregulation between the CR and NO-CR groups (Table 1). At 22 weeks, the global effects of CR on protein FSRs were reversed in the absence of NO. The comparison showed that 78.1% of proteins were downregulated under CR, while 33.7% were downregulated in the NO-CR group (*P* = 1.5 × 10^–8^).

### Exploring the role of NO in physiological outcomes.

**Figure 6:**
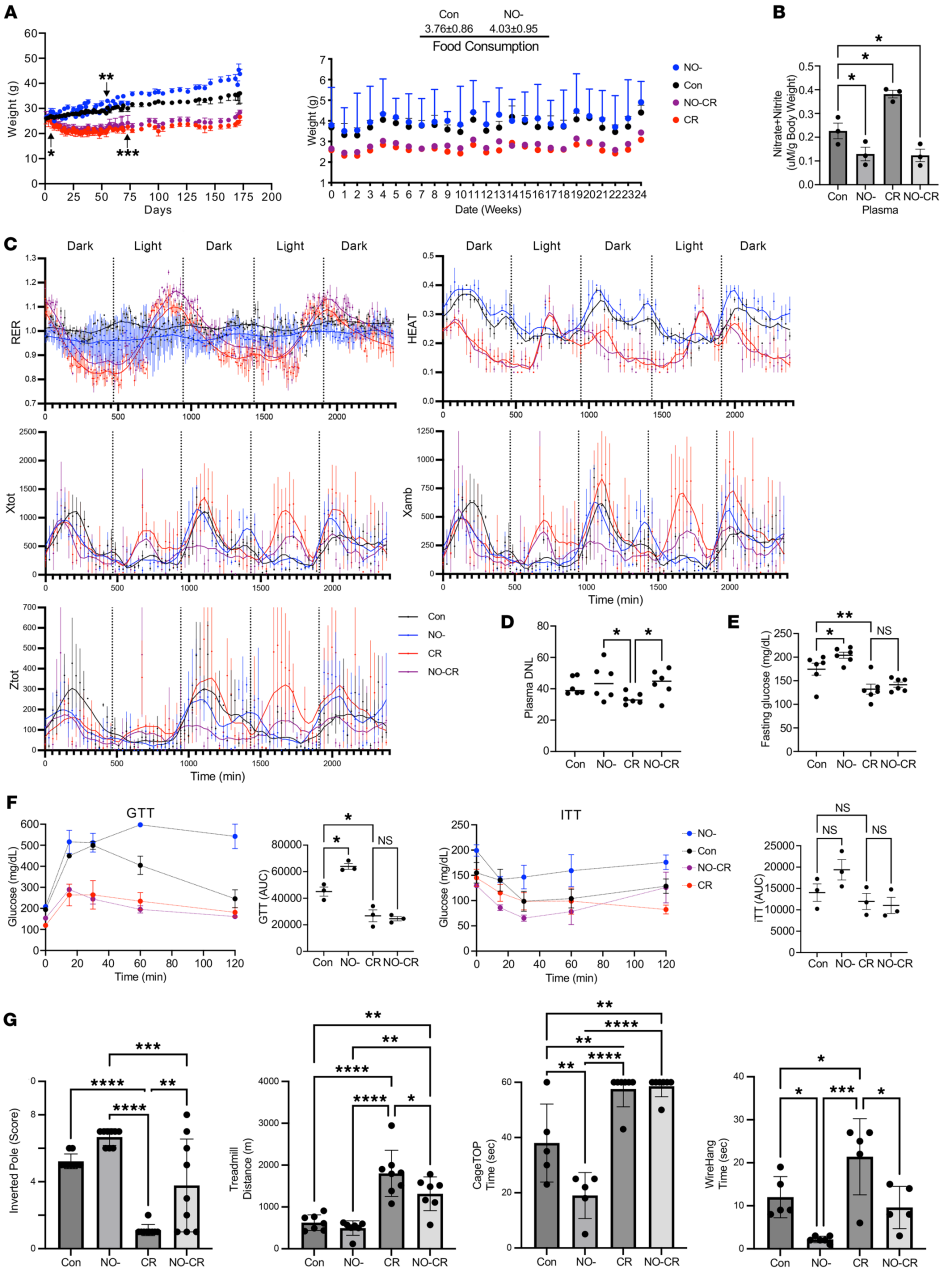
Experimental design for the physiologic effects of CR in iNOS-KO (NO^–^) mice. (**A**) Mice were single-caged, and their body weights and food consumption were measured throughout the intervention period. Mice under CR consumed 30% less food than did Con mice. Significance is indicated by an arrow; *P < 0.05 between Con and CR, **P < 0.05 between Con and NO^–^, and ***P < 0.05 between CR and NO-CR. *P* values were determined using 1-way ANOVA for all comparisons. (**B**) Plasma nitrate+nitrite concentrations (μM) normalized to mouse body weights (g) to represent NO production. *P < 0.05, by 1-way ANOVA. (**C**) Metabolic cage (CLAMS) assessment of mice (*n* = 6–9 per group). Animals were placed in a CLAMS, and their metrics were recorded for 3 nights (dark) and 2 days (light). Curves were smoothed to the sixth polynomial order. (**D**) Free plasma palmitate was esterified and analyzed for mass isotopomer abundances by GC-MS. *P < 0.05, by 1-way ANOVA with Fisher’s LSD test. (**E**) Fasting blood glucose levels (*n* = 6 per group). *P < 0.05 and **P < 0.005, by 1-way ANOVA. (**F**) GTTs and ITTs were performed under fasting conditions. The levels of glucose in the blood of mice fasted for 6 hours were measured over time after i.p. insulin injection for the ITT (*n* = 3 per group). Blood glucose levels after an i.p. glucose load (GTT) were measured in mice fasted overnight (*n* = 3–4 per group). *P < 0.05, by 1 way ANOVA for AUC analysis. (**G**) Behavioral and performance test results. Mice were subjected to treadmill exercise until exhaustion. Cage top and wire-hang tests were conducted for up to 60 seconds. The inverted pole test, assessing motor function, was scored by the time required to turn downward. *P < 0.05, **P < 0.005, ***P < 0.0005, and ****P < 0.00005, by 1-way ANOVA. HEAT, heat production measured as kcal/hr; Ztot, vertical plane; Xtot, horizontal plane total movement; Xamb, ambulatory movement.

We then asked whether there is a functional role for NO in the physiological outcomes showing a response to CR. We first compared body weights (Figure 6A) measured over time. Previous studies have shown that iNOS-KO mice exhibit significant increases in body weight and fat content, a reduced respiratory exchange ratio (RER), lower carbon dioxide production (VCO_2_), and decreased heat production compared with Con, suggesting an impaired metabolic rate and altered substrate utilization (48). We observed significant body weight differences between the Con and CR groups during the first week of intervention (Figure 6A). After 7 and 10 weeks of CR, the NO-CR mice were significantly heavier than the CR group. Measurements of food consumption showed no significant differences (Figure 6A).

To assess NO production, we measured plasma nitrate and nitrite levels, normalized to mouse body weight, as a proxy (20, 49, 50). These stable byproducts of NO are a preferred measurement because of the short lifespan of NO (51). We observed lower nitrate-plus-nitrite (nitrate+nitrite) levels in the NO^–^ groups compared with the Con group, under both CR and ad-lib conditions. In addition, CR significantly increased nitrate+nitrite levels (Figure 6B).

To evaluate energy usage and behavioral movement, we utilized metabolic cages (comprehensive lab animal monitoring system [CLAMS]) (refer to Supplemental Figure 2 for metabolic cage outcomes) and measured the RER, heat production measured as kcal/hr (HEAT), and movement scores (vertical plane [Ztot], horizontal plane total movement [Xtot], and ambulatory movement [Xamb]) (Figure 6C). As we described previously, CR induces a wide circadian shift in the RER related to the meal-feeding pattern of food intake (the mouse’s entire daily ration of food is ingested over the 2–3 hours each day rather than being spread out over 24 hours; ref. 52) compared with Con. CR mice displayed a significantly lower AUC of RER values during the dark cycle (*P* < 0.05), which is consistent with predominantly fat utilization after the first few hours of food intake (52). This effect, however, was not observed in the NO-CR group (Figure 6C). We also measured de novo lipogenesis (DNL) (Figure 6D), as previously described (36, 53, 54). CR mice displayed a trend toward lower DNL values compared with Con mice (*P* < 0.07) in the measured period. Notably, NO-CR mice exhibited a statistically significantly higher DNL compared with CR mice (*P* < 0.05).

To assess insulin sensitivity, we measured fasting plasma glucose levels (Figure 6E) and conducted glucose tolerance tests (GTTs) and insulin tolerance tests (ITTs) (Figure 6F). Both fasting plasma glucose levels and AUC analysis from the GTTs showed statistically significantly higher glucose concentrations in the NO^–^ group than in the Con group (*P* < 0.05), confirming previous observations that iNOS-KO mice exhibit altered glucose homeostasis (48). However, CR effectively mitigated these effects, with no significant differences observed between the CR and NO-CR groups in either test. The ITT, performed after a 6-hour fast, revealed no statistically significant changes across any group by AUC analysis.

Several behavioral and performance tests (Figure 6G) were conducted. (a) The inverted pole sensorimotor test (32, 55, 56) was performed and involves placing the mouse with its head up on top of a pole and measuring the time it takes to orient the body downward and descend. The scoring was based on time, where a higher score indicates a worse outcome. Our results indicated that both the Con and NO^–^ groups, under ad-lib conditions, had similarly lower performance scores (Con mean: 5.22, NO^–^ mean: 6.67) compared with the CR groups. CR mice attained the highest performance score, with a mean score of 1.1. However, this improvement was attenuated in the NO-CR group, which had a mean score of 3.78. (b). The treadmill test (representing fatigue-like behavior [ref. 57] and aerobic fitness [refs. 32, 58]) was performed by placing mice on a treadmill and gradually increasing the speed until exhaustion. Both the Con and NO^–^ groups, under ad-lib conditions, had similarly lower performances (Con group mean: 625 meters traveled, NO^–^ group mean: 495.1 meters traveled) compared with the CR groups. CR mice had the highest performance, with a mean distance of 1,803 meters traveled. However, this improvement was reduced in the NO-CR group of mice, which traveled a mean distance of 1,315 meters before reaching exhaustion. The mean difference between the CR and NO-CR groups was 488 meters (*P* = 0.0368). (c) The cage top suspension test was performed to assess sensorimotor reflexes and motor response by measuring the latency of a mouse to fall, for up to 60 seconds, while suspended upside down from a wire cage top (32, 59, 60). Our results indicated that the NO^–^ group had the lowest performance, with an average time of 19.0 seconds before falling. The CR and NO-CR groups exhibited the highest performance of remaining on the wire cage top, going beyond the allocated 60 seconds. (d) The wire-hang test involves a method used to assess muscle performance (32, 61) by measuring the time it takes for the mouse to lose its grip on a wire, up to 60 seconds. Our results indicated that the NO^–^ mice had the lowest performance, with an average time of 2.167 seconds before falling. The Con mice had an average time of 12.0 seconds before falling. Again, the CR group had the best performance, with an average time of 21.4 seconds before falling, and this effect was attenuated in the NO-CR group, in which the average time before falling decreased to 9.6 seconds.

**Figure 7:**
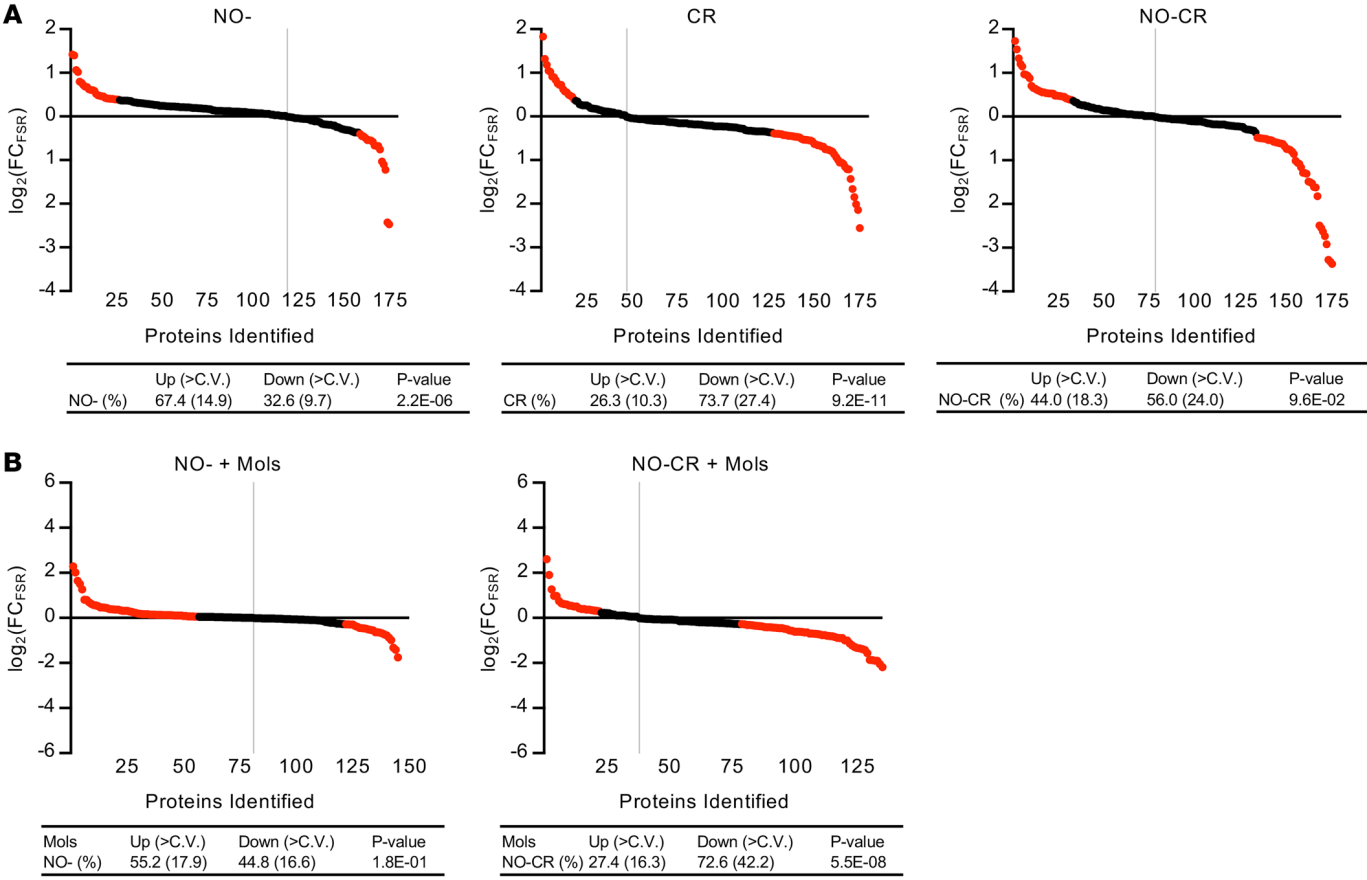
Effects of iNOS KO, pharmacological rescue, and CR on hepatic proteome FSRs. (**A**) FSR values were obtained for the liver proteome at 6 weeks of intervention for Con, iNOS-KO (NO^–^), and CR iNOS-KO (NO-CR) groups. Dots along the line represent identified proteins sorted from the highest FSR to the lowest FSR for the group by the log_2_ FC compared with the ad-lib Con group. Comparisons were done for all groups. Tables represent the binomial distribution of proteins with an increased (Up) or decreased (Down) FSR. In red are the proteins whose change was greater than the CV. *P* values in the tables were determined using a 2-tailed binomial distribution. (**B**) FSR values of the liver proteome at 6 weeks of CR. Included in this experiment were rescue experiments with Mols, given in the drinking water daily to rescue the effects of CR on proteome regulation in NO^–^ mice. Mols is a potent donor of NO metabolized in the liver. NO^–^ mice were given Mols in the drinking water for the same length of intervention as CR (6 weeks).

*Can the reversal of CR effects on hepatic proteome fluxes by iNOS KO be overcome?* Focusing more closely on the period that included the transition between day 25 and day 30, we conducted a 6-week CR intervention experiment to determine whether the prevention of CR effects on hepatic proteome fluxes by NO^–^ can be rescued (Figure 7). We administered molsidomine (Mols), a NO donor that is metabolized by hepatic esterases. This is a liver-specific metabolic process that converts Mols into its active metabolite, SIN-1, which subsequently releases NO (62–64). The inclusion of Mols in the experiment addressed whether the effects of CR on proteome regulation in the NO^–^ mice were mitigated by NO replacement. Considering the short half-life of Mols in plasma of approximately 1–2 hours, we administered Mols continuously via the drinking water. Mols was administered at a dose of 120 mg/L (65, 66) and was maintained for the duration of the 6-week CR intervention.

Several key observations are apparent (Figure 7A). First, at 6 weeks after the start of the study (which began for mice at 6 weeks of age), the NO^–^ group had a significantly higher fraction of proteins with increased FSRs (67.4% of proteins). When considering only those proteins above the CV, 14.9% showed increased FSRs, whereas 9.7% had a decrease in FSRs. Within the CR group, the majority of proteins (73.7%) showed slower FSRs, and considering those above the CV, 27.4% were downregulated, with 10.3% showing upregulation. In the NO-CR group, the effects of CR were lessened significantly (Table 2), going from 73.7% under CR to 56.0% in the NO-CR group (*P* = 0.049). These findings confirmed our observations at 10 and 22 weeks of CR (Table 1).

In the Mols rescue group (NO^–^+Mols, Figure 7B), the trends toward higher FSRs with NO^–^ alone were significantly diminished, dropping from 67.4% of proteins with higher FSRs in the NO^–^ group alone compared with 55.2% after Mols administration. Notably, the effects on protein FSRs in the NO-CR group were rescued after daily administration of Mols for the 6-week intervention (Table 2). The effect of CR alone on reduced FSRs was maintained in the NO-CR+Mols group at 72.7% (*P* = 0.457).

These results demonstrate that the administration of Mols to NO-CR mice can restore the effects of CR alone on protein FSRs.

### Reversing the effects of CR through pharmacological inhibition of iNOS.

**Figure 8:**
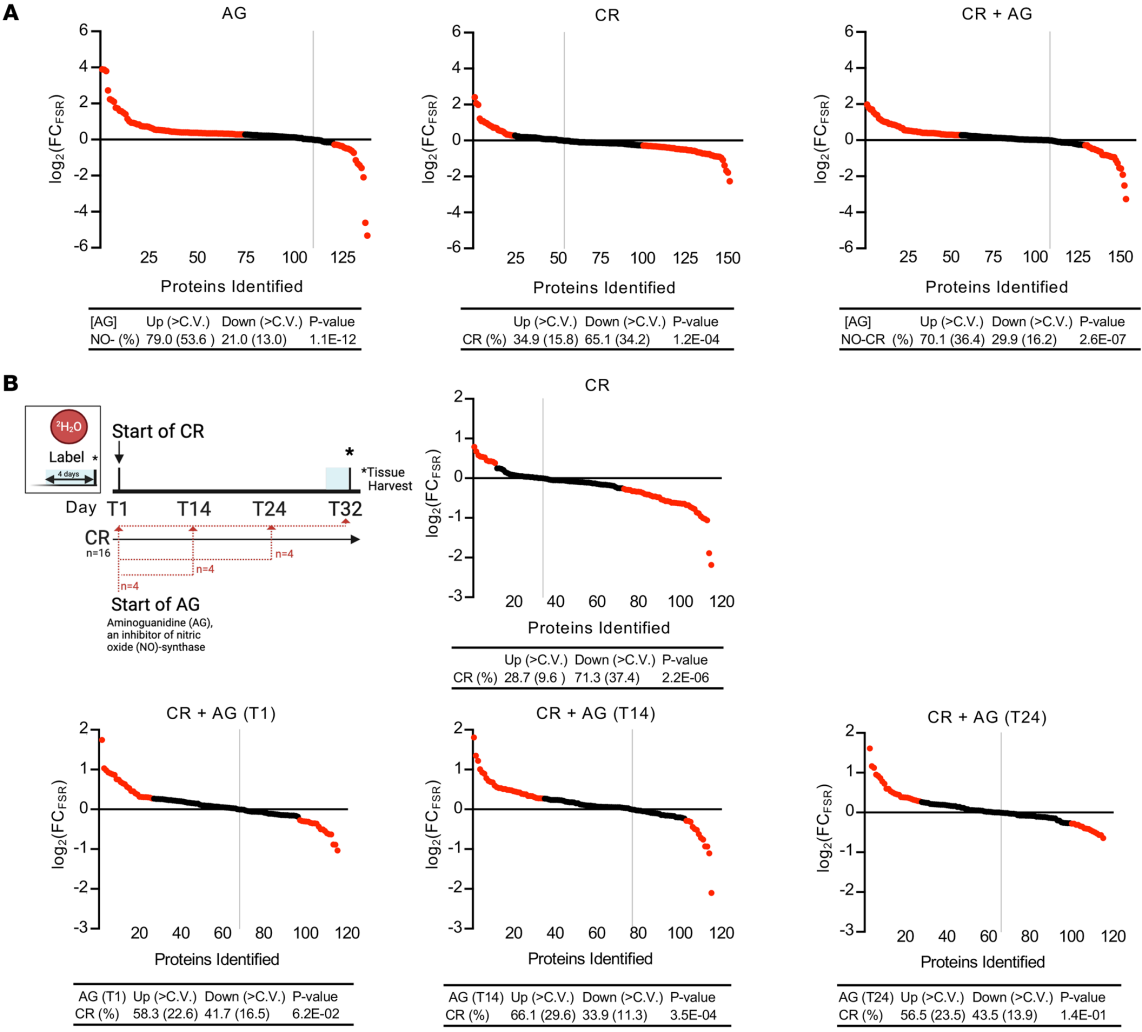
Pharmacological inhibition of NO by AG in relation to the threshold event of CR. A pharmaceutical approach was tested. AG is a selective inhibitor for all NOS enzymes. (**A**) Mice were given AG in the drinking water daily for 6 weeks. FSR values for hepatic proteins measured in all 4 groups were compared. *P* values in the tables were determined using a 2-tailed binomial distribution. (**B**) Experimental design for the time-course study of the threshold transition event. Mice underwent CR for 32 days, and AG was given in the drinking water daily starting at day 1 (T1), day 14 (T14), or day 24 (T24) to inhibit NOS. CR mice were compared with ad-lib Con mice. Mice with CR plus AG treatment were compared with mice subjected to CR alone without an inhibitor for the full 32 days. These findings show that inhibition of NO by AG reversed the effects of CR on hepatic proteome fluxes, whether during the entire period of CR, the last 18 days of CR, or during the 8-day period immediately preceding day 32 (the “threshold” or transition period).

Next, we explored the inhibition of iNOS pharmacologically with aminoguanidine (AG), a highly selective inhibitor of iNOS, during the first 6 weeks after initiating CR. AG has been shown to be more than 50 times more effective at inhibiting the enzymatic activity of iNOS compared with eNOS or neuronal NO synthase (nNOS) (67–70). The experimental mice were placed on 6 weeks of CR, beginning at week 6 of age. AG was administered by dissolving it in the drinking water at a concentration of 2 mg/mL (71, 72) and was provided daily to mice in both the CON and CR groups (Figure 8A).

We observed 79.0% of proteins with higher FSRs in the AG-treated group compared with the Con group. Notably, 53.6% of the proteins were upregulated beyond the CV, which aligns with our findings in NO^–^ genetic KO mice. In the CR group, a total of 65.1% of protein fluxes were downregulated, with 34.2% of them falling above the CV threshold.

The effects of CR were reversed by AG administration during the 6-week period of CR: 70.1% of proteins had higher FSRs, with 36.4% exceeding the CV threshold. These findings are consistent with our previous observations with the genetic KO, demonstrating that in the absence of NO, the effects of CR on slowing down protein turnover rates were either markedly weakened, as seen at 6 weeks of CR in the genetic NO-CR group (Figure 7A) or completely reversed, as observed at 22 weeks of CR in the genetic NO-CR group (Figure 5B) and at 6 weeks of CR in the pharmacological AG-CR group (Figure 8A).

### Investigating the role of NO in the transitional event during CR.

In order to assess in finer detail the effects of NO on the transition that occurs between day 25 and day 30 of CR (Figure 3), we conducted a time-course study with AG administration (Figure 8B). Mice were placed on a CR regime for 32 days, and AG was administered daily by dissolving it in their drinking water at 2 mg/mL, starting at different time points — day 1 (T1), day 14 (T14), or day 24 (T24) — and continuing until study termination at day 32.

First, we compared the effects of CR with the ad-lib Con group at day 32. We observed that 71.3% of proteins had lower FSR values, with 37.4% exceeding the CV threshold. The CR+AG groups over the 32-day time course were then compared.

The time-course study revealed that inhibition of NO by AG effectively reversed the effects of CR on hepatic proteome fluxes when initiated at the start of the experiment on day 1 (T1). Hepatic proteome flux analysis showed that 58.3% of proteins had a higher FSR, with 22.6% being above the CV threshold, when compared with CR alone. This effect was also observed when AG was started at day 14 of CR (T14), when 66.1% of proteins had higher FSR values, with 29.6% of these being above the CV threshold. Most remarkably, starting the inhibition of NO by AG at day 24 (T24), one day before the previously observed transition period, blocked the effect of CR alone, with 56.5% of proteins having higher FSR values, and with 23.5% of these being above the CV threshold.

Accordingly, reversal of the effects of CR on the hepatic proteome was observed whether AG was administered throughout the entire duration of CR or only during the 8-day transition period preceding day 32. This highlights that NOS inhibition during this discrete time window of CR prevents the downregulation of hepatic proteome fluxes.

## Discussion

Protein replacement rates have emerged as a promising focus for aging and lifespan extension research (2, 5, 30). Understanding the underlying mechanisms of these interventions is crucial. However, both the time frame involved in the beneficial effects of these interventions and the signaling mechanisms involved remain unclear.

Advances in proteome dynamics using heavy isotope labeling followed by LC-MS/MS analyses have facilitated the understanding the suppression of protein synthesis rates by CR. We (2, 5) and others (Rabinovitch and colleagues, refs. 7–9) have reported consistently lower protein turnover rates in CR, although other authors have also reported that CR increases protein synthesis as a mechanism of lifespan extension (73–77).

We present here results using heavy water labeling and combinatorial analyses (MIDA) (27) to define the detailed time course of changes in hepatic proteome-wide protein fluxes and the role of NO. We found modest, not consistently substantial, hepatic protein turnover changes between the Con and CR groups during the early stages of CR (days 9–25). However, starting from days 25–32, we observed a striking inflection toward systematically slower protein turnover rates, which persisted or intensified as the period of CR progressed (days 43–170). These findings suggest that comprehensive remodeling of hepatic protein fluxes evolves within approximately 30 days of CR and that the period between days 25 and 32 may reveal underlying signals of this adaptation and represent a discrete period for therapeutic interventions.

A hallmark of aging is a loss of the ability to maintain proteostasis, leading to a wide array of dysregulated metabolic processes that affect physiologic and health outcomes (4, 78–80). CR and CR mimetics have been shown to preserve cellular proteostasis and ensure proper protein quality control through mechanisms such as chaperone-mediated autophagy (81). Although the causal processes that underlie CR-induced lifespan extension in rodents remain unproven, a reduction in protein turnover during CR may contribute in several ways. Reducing the metabolic load on cells, the demands for energy, and the accumulation of misfolded or damaged proteins may be critical factors in promoting cellular health and longevity. It has been suggested that the translation capacity of the cell can change during CR, and mitochondrial biogenesis during CR has been a key focus of study. For example, Nisoli et al. (82) reported that mice on a 30% CR diet for 3 months exhibited increased mitochondrial biogenesis, indicated by higher mitochondrial DNA content, elevated expression of the mitochondrial biogenesis regulators PGC-1α, NRF-1, and Tfam, and higher levels of cytochrome c oxidase and cytochrome C (82). However, our findings here are not consistent with increased mitochondrial biogenesis and protein synthesis. Instead, we observed here and previously (2, 5) that most gene ontologies, including those for mitochondrial proteins, exhibit globally slower protein turnover rates in liver during CR.

We examined whether CR modulates NO-dependent proteins under endogenous or pharmacologically provided NO. At both 10 and 22 weeks of CR, 78% of proteins showed consistent regulation in the hepatic proteome (Supplemental Figure 5A). Without NO, CR resulted in no significant differences compared with the Con (Supplemental Figure 5B). Pharmacological restoration of NO with Mols rescued the CR effects, with only 12.4% of proteins remaining inconsistent between CR and NO-CR+Mols (Supplemental Figure 5C).

Interestingly, contrary to some reports (83, 84), changes in protein turnover rates during CR did not occur at the transcriptional/mRNA level. Instead, CR increased protein half-lives without clear corresponding changes in gene expression. These findings suggest that the important signals driving protein changes during CR are primarily controlled at the posttranscriptional/translational level.

In terms of function, CR enhances NO bioavailability by converting arginine to citrulline through NOS activity (17, 19, 85, 86). Moreover, a link between NO production and lifespan has been observed in living organisms, as bacterial NO extended the lifespan of *C*. *elegans* (10). Indeed, NO appears to play a key role as a regulator of many CR-linked pathways that regulate proteostasis (87–90), which motivated NO as a target here. Previous studies have shown that NO can inhibit translation both in vitro and in vivo, leading to the stalling and collision of ribosomes (91, 92). This activates several ribosomal stress surveillance pathways, including the ribosome quality control mechanisms. NO-induced ribosome collision enhances GCN2-mediated phosphorylation of eIF2α, which collectively may contribute to the inhibition of global protein synthesis. These findings align with our observations that increased NO bioavailability during CR is associated with reduced protein synthesis rates and that disrupting this process can affect both protein translation processes and protein surveillance systems (93).

Hepatic proteome flux analyses revealed that many proteins in NO^–^ mice had higher turnover rates than did those in Con mice. Conversely, the ability of CR to slow down the hepatic proteome was markedly impaired in NO^–^ mice. Protein turnover was consistently higher in the absence of NO. For example, beyond 30 days of CR in every experiment performed, CR led to a reduction in protein turnover, but the majority of proteins had higher FSRs in NO-CR mice compared with mice subjected to CR alone.

Administering the NO-generating drug Mols during CR rescued the proteome-wide effects of CR in NO-CR mice, with 72.6% of proteins exhibiting downregulation. These findings highlight the regulatory role of NO in modulating hepatic proteome fluxes during CR and identify potential pharmacologic strategies targeting NO metabolism for CR mimetics.

When examining the transitional event of CR over a 32-day period, we discovered that pharmacological inhibition of NO with AG at various time points reversed the effects of CR on hepatic proteome fluxes, regardless of whether the inhibition was applied throughout the entire 32-day CR period, was initiated the last 18 days of CR, or began specifically at day 24 of CR. Thus, even when it was present only during the final 8 days, AG effectively prevented the slowing of protein turnover by CR.

In addition, the effects of NO on mouse physiology revealed distinctive effects of this signaling molecule, including on body composition, behavior, and physical performance. iNOS-KO mice had previously been shown to have altered glucose homeostasis toward insulin resistance (48). Concordant with these findings, the NO^–^ group showed increased glucose concentrations in plasma both during fasting and after a GTT, although no difference was observed between the CR and the NO-CR groups.

The effects of CR were consistently beneficial in terms of physiology and behavior, while the NO^–^ and NO-CR groups consistently showed less favorable outcomes. In the inverted pole test, mice in the Con and NO^–^ groups exhibited similar performances under ad-lib conditions, but mice in the CR group performed the best, while the NO-CR group showed limited improvement. In the treadmill test, mice in the Con and NO^–^ groups also had lower performances compared with the CR groups, with the CR group displaying the highest performance and the NO-CR group showing lower performance. In the Cage top test, the NO^–^ group had the lowest performance, while the CR and NO-CR groups had higher performances. Similarly, in the wire-hang test, the NO^–^ group had the lowest performance, and the CR group had the highest performance. Overall, mice in the CR groups consistently outperformed the Con and NO^–^ groups, while mice in the NO-CR group had somewhat lower performances compared with those in the CR group.

Rabinovitch’s group, in a series of important studies (7–9), utilized ^2^H_3_-leucine labeling followed by LC-MS/MS to examine the effect of CR on proteome regulation and age-related cardiac dysfunction. Their studies demonstrate that CR enhances protein homeostasis in the heart by improving intracellular calcium handling and autophagy via mTOR suppression. They found that 10 weeks of CR suppressed protein synthesis rates, and 10 weeks of either CR or rapamycin treatment, initiated at an older age, rejuvenated the aging mouse heart by altering proteome turnover and remodeling. These findings align with our key result of increased protein half-lives in liver in response to CR. While Rabinovitch’s group primarily focused on cardiac tissue, they observed similar increases in protein half-lives in the liver after CR, consistent with our findings.

In summary, we focused here on hepatic protein synthesis rates in relation to CR and NO signaling. Our findings show that substantial changes in global protein turnover rates occurred around days 25–32 of CR and persisted through at least approximately 6 months of CR. These changes were not accompanied by corresponding alterations in gene expression. Our data show a crucial role of NO in CR for protein synthesis pathways and for maintaining physiologic improvements. Beneficial effects of CR on physiology and behavior were consistently observed, while a reduction of NO genetically or pharmacologically disrupted the ability of CR to slow down protein turnover rates and resulted in worse physiologic performance. Pharmacologic replacement of NO rescued CR effects. Moreover, pharmacological inhibition of NO even during the final 8 days of a 32-day CR intervention effectively reversed the effects of CR on protein turnover rates. These findings underscore the importance of NO signaling in the proteostatic effects of CR, provide a narrow time window to target CR-mimetic interventions, and may offer valuable insights into underlying mechanisms of aging and longevity.

## Methods

### Sex as a biological variable.

Our study examined male mice on the basis of previous studies analyzing CR FSRs, which were also conducted using male mice. Since our study followed these previous studies, using male mice provided consistency and comparability in results. It is currently unknown whether the findings are relevant for female mice, and future studies are necessary.

### Mouse models.

Six-week-old male C57Bl/6J Con mice and B6.129P2-Nos2tm1Lau/J–transgenic mice (NO^–^) were purchased from The Jackson Laboratory (stock no. 002609). All mice were housed individually and maintained under temperature- and light-controlled conditions (12-hour light/12-hour dark cycle, lights on at 0700 hours and off at 1900 hours). Mice were randomly assigned to the ad-lib or CR group. Mice in the AL group were provided unrestricted access to the NIH41 diet (diet no. 58YP, TestDiet). CR mice were provided an NIH41-fortified diet (diet no. 5TPD, TestDiet) at 30% less calories than the AL group, with weekly adjustments to maintain body weight at approximately 75% of the AL group average. Food was provided daily at 1200 hours. All mice were kept on their diets for a total of 9–170 days. The body weight of each mouse was measured 3 times per week. Mice were labeled with heavy water 4 days before sacrifice. The proteomics approach presented here was based on a previous publication (2).

### GTTs and ITTs.

For GTTs, mice were fasted overnight, and glucose (2 mg/g) was administered i.p. For ITTs, mice were fasted for 4  hours, and insulin (0.75 U/kg) was administered.

### In vivo respirometry.

Whole-body energy expenditure (VO_2_, VCO_2_) was recorded at the indicated environmental temperatures using a CLAMS (Columbus Instruments).

### Heavy water–labeling protocol.

To measure the rates of in vivo protein replacement, mice were labeled with an i.p. injection of 100% ^2^H_2_O 4 days prior to sacrifice. Mice were provided free access to 8% heavy water as drinking water during the labeling period, as previously described (2, 5, 27).

### Blood, plasma, and tissue collection.

Upon completion of each study, the mice were anesthetized under 3% isoflurane, and blood was collected via cardiac puncture, followed by cervical dislocation and tissue collection. Following centrifugation of the blood, plasma was collected and stored at –80°C. Upon dissection, the liver was cut into several small pieces (~20–100 mg) that were then flash-frozen in liquid nitrogen for further analysis.

### Calculation of DNL.

The measurement of newly synthesized palmitate formed during ^2^H_2_O labeling period was assessed using a combinatorial probability model of polymerization biosynthesis, as described previously (53, 54, 94, 95). MIDA, along with body ^2^H_2_O enrichment, was used to determine the theoretical maximum enrichment of palmitate during the period of label exposure (27, 54, 94, 95) and to calculate fractional contributions from the DNL pathway.

### NO assessment.

To assess NO levels in plasma, NO_2_/NO_3_ levels were measured using a commercial kit from Cayman Chemical (catalog 780001). The Griess reaction was used according to the provided protocol. The values obtained from the Griess assay represent the combined amount of nitrite and nitrate (NO) (96, 97). Data were normalized to body weight.

### Measurement of ^2^H_2_O enrichment in body water.

Enrichment of ^2^H_2_O in body water (blood) was measured via chemical conversion to tetrabromoethane by gas chromatography–mass spectrometry (GC-MS) analysis as previously described (2, 5, 30). Body water ^2^H_2_O enrichment values (*p*) were used to calculate the fractional synthetic rate (*f*) of peptides as previously described (2, 5, 27).

### Preparation of liver samples for LC-MS/MS proteomics analysis.

Frozen livers from mice labeled with heavy water were prepared and treated as previously described (2, 5, 27). Briefly, livers were homogenized in lysis buffer, sonicated, and centrifuged. Protein concentrations were determined by Bicinchoninic acid (BCA) assay (Pierce, Thermo Fisher Scientific). Aliquots (100–250 μg) were reduced with tris(2-carboxyethyl)phosphine and alkylated with iodoacetamide. Tryptic peptides were then prepared as previously described (2, 5, 27).

### LC-MS/MS analysis.

The LC-MS/MS method utilized in this study followed the protocols routinely used in our laboratory. Trypsin-digested peptides were analyzed using an Agilent 6550 Q-TOF mass spectrometer with a 1260 Chip Cube nano ESI source (Agilent Technologies). Peptides were separated chromatographically on a Polaris HR chip (Agilent Technologies), and mobile phases consisted of acetonitrile and formic acid in deionized water. Specific gradient and acquisition parameters for the LC-MS/MS procedures can be found in our previously published work (2, 5, 36, 93). Acquired spectra were extracted and searched using Spectrum Mill Proteomics Workbench software and the UniProtKB/Swiss-Prot mouse protein database. Detailed sample preparation, chromatographic conditions, and data analysis protocols were previously described (5, 28, 36, 93, 98).

### Proteome flux calculations.

Protein measurements with fewer than 2 peptide spectra per protein were excluded. Fractional replacement calculations, as previously described (2, 5, 27), were conducted using in-house software to predict peptide isotope enrichments based on precursor body water enrichment (*p*) and the number of C-H positions (*n*) incorporating H and 2H. Incorporation of 2H into tryptic peptides was assessed via the isotope envelope M0–M3. Fractional synthesis ratios were calculated as the excess percentage of M0 (EM0) over the maximal absolute EM0 at measured body water enrichment. Data handling was done using Microsoft Excel, yielding FSR data at the protein level. FSR data at individual time points (weeks) are reported as cumulative values (percentage of protein newly synthesized over the labeling period).

### AG administration.

AG was administered to mice as previously described (71, 72). In short, AG was purchased from MilliporeSigma (no. 19266598). AG was dissolved in the same drinking water used for the animal colony at 2 mg/mL and sterile filtered through a 0.22 μm disposable filters (MilliporSigma, Millex-GV) before administration. The solution was made fresh and given to mice in their drinking water bottles daily. Bottles with AG were changed every day for the duration of the experiment.

### Mols administration.

Mols was administered to mice as previously described (65, 66). In short, Mols was purchased from MilliporeSigma (catalog M2901). Mols was dissolved in the same drinking water used for the animal colony at 120 mg/L and sterile-filtered through a 0.22 μm disposable filter (MilliporeSigma, Millex-GV) before administration. The solution was made fresh and given daily to mice in their drinking water bottles. Bottles with Mols were changed every day for the duration of the experiment.

### Euthanasia.

Animals were anesthetized with isoflurane and euthanized by cardiac puncture.

### Liver tissue RNA-Seq.

Total RNA was isolated from frozen liver samples using QIAzol lysis reagent and an RNeasy Mini Kit with the RNase-free DNase Set (Qiagen), as previously described (99). RNA quantity and quality were assessed using a Qubit fluorometric assay and Eukaryote Total RNA Pico (Agilent Bioanalyzer 2100), respectively. Library preparation and sequencing (100 bp pair-end reads) were performed on a single lane of Illumina HiSeq 4000 at the Vincent J. Coates Genomics Sequencing Laboratory (UC Berkeley). Raw files were processed with CASAVA 1.8.2 (Illumina) to generate fastq files and analyzed with FastQC, yielding high-quality scores. Fastq files were uploaded to the Galaxy project portal (https://usegalaxy.org/) for analysis. Reads were mapped to the mouse reference genome (mm10) using BWA, and differential gene expression was determined using DESeq2.

Additional details can be found in the Supplemental Methods.

### Statistics.

Data are reported as the mean ± SEM unless otherwise noted. A *P* value of 0.05 or less was considered statistically significant. Data obtained from studies conducted in mice were analyzed using MetaboAnalyst (100), GraphPad Prism (version 9.0, GraphPad Software), InfernoRDN (https://omics.pnl.gov/software/infernordn) Windows application (version 1.1), gene set enrichment analysis (GSEA) software and Molecular Signatures Database (MSigDB), visualization by SRPLot (101), and Real Statistics Resource Pack in Excel (version 16). Independent and paired 2-tailed Student’s *t* tests, 2-way ANOVA, and 1-way ANOVA were performed where appropriate. Tukey’s or Šidák’s multiple-comparison tests were applied for secondary post hoc analyses where appropriate. Significance for other analyses is denoted as *P < 0.05, **P < 0.005, ***P < 0.0005, and ****P < 0.00005. AUC analysis was performed for significance using 1-way ANOVA (*P < 0.05). Fisher’s exact test and binomial distribution 2-tailed *P* values were applied where relevant. The log_2_ FC and CV were calculated where appropriate. Comparisons and replicate numbers are listed in each figure legend.

### Study approval.

All animal experiments were conducted according to the animal use protocol, approved by the IACUC of UC Berkeley.

### Data availability.

RNA-Seq data have been deposited in the NCBI’s Gene Expression Omnibus (GEO) database (GEO GSE274644). Raw data used to generate the descriptive statistics presented in this manuscript are provided in the Supporting Data Values file. This manuscript does not report original code. Additional information, resources, and reagents required for reanalysis of the data reported in this study are available from the corresponding author.

## Author contributions

HHP, EC, and AC conducted the mouse experiments. HHP, EC, and AC performed sample analysis. HHP and HM conducted the proteomics experiments. HHP and MKH analyzed and interpreted the data. HHP and MKH designed the experiments and wrote the manuscript. HHP and MKH designed and MKH supervised the studies and obtained funding for the work. MKH is the guarantor of this work and, as such, had full access to all the data in the study and takes responsibility for the integrity of the data and the accuracy of the data analysis. All authors critically reviewed and edited the manuscript.

## Funding support

This work is the result of NIH funding, in whole or in part, and is subject to the NIH Public Access Policy. Through acceptance of this federal funding, the NIH has been given a right to make the work publicly available in PubMed Central.

- NIH grant T32HL130357.
- NIH Instrumentation Grant S10OD018174 (in support of the Vincent J. Coates Genomics Sequencing Laboratory at UC Berkeley).

## Supplementary Material

Supplemental data

Supporting data values

## Figures and Tables

**Table 2:**
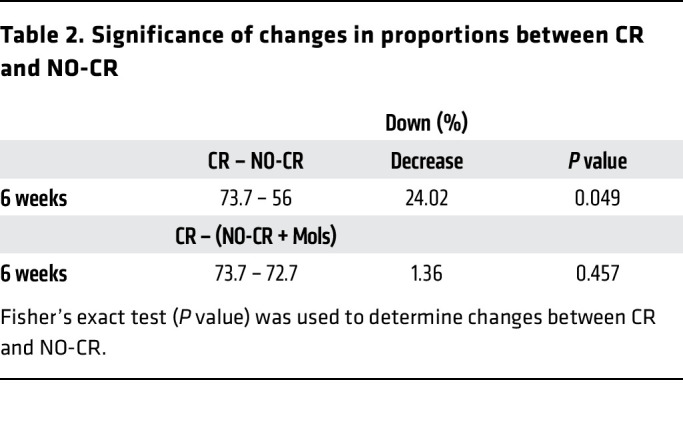
Significance of changes in proportions between CR and NO-CR

**Table 1:**
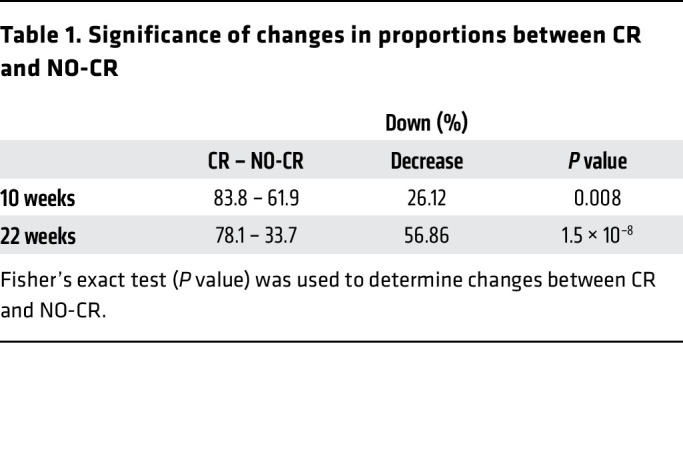
Significance of changes in proportions between CR and NO-CR

## Version 1 01/02/2026

Electronic publication
