# Supplementary material for: Nitric oxide required for transition to slower hepatic protein synthesis rates during long-term caloric restriction
Source: J Clin Invest. 2026 Jan 2;136(1):e189798. doi: 10.1172/JCI189798 (PMC12721896; doi:10.1172/JCI189798)
Supplement: Supplemental data [file jci-136-189798-s202.pdf]

## Methods

### Within proteome absolute synthesis (WPAS) measurement

Label-free quantitative proteomics was used to calculate within proteome absolute synthesis (WPAS) rates. Signal intensities of unlabeled peptides were log2 transformed and mean-centered to account for variations in sample loading and instrument variability. Median normalization was applied to adjust for systemic differences among samples. Individual values were transformed using log base 10 and scaled to mean center by adjusting each feature with a scaling factor based on the dispersion of the variable.

Quality control involved creating a Pearson cross-correlation matrix to examine peptide-level intra-group and inter-group variability. Peptides were aggregated into parent proteins using the top 30% most intense peptides, excluding proteins containing only a single peptide from further analysis. A second Pearson cross-correlation matrix was created to examine protein-level intra-group and inter-group variability and clustering. Signal intensities of experimental groups were normalized against the age-matched control group means. All calculations, plots, and analyses were performed using Inferno for Proteomics published by the Pacific Northwest National Laboratory (PNNL).

WPAS values were derived by calculating the label-free normalized signal intensity quotient (Q) for each protein by correcting individual signal intensities against the control group mean. WPAS for each protein was determined by multiplying the fractional synthesis rates (f) with the label-free values (Q) using the formula: $WPAS = f * Q$. This calculation represents the relative mass of newly synthesized protein within the proteome.

## Figures

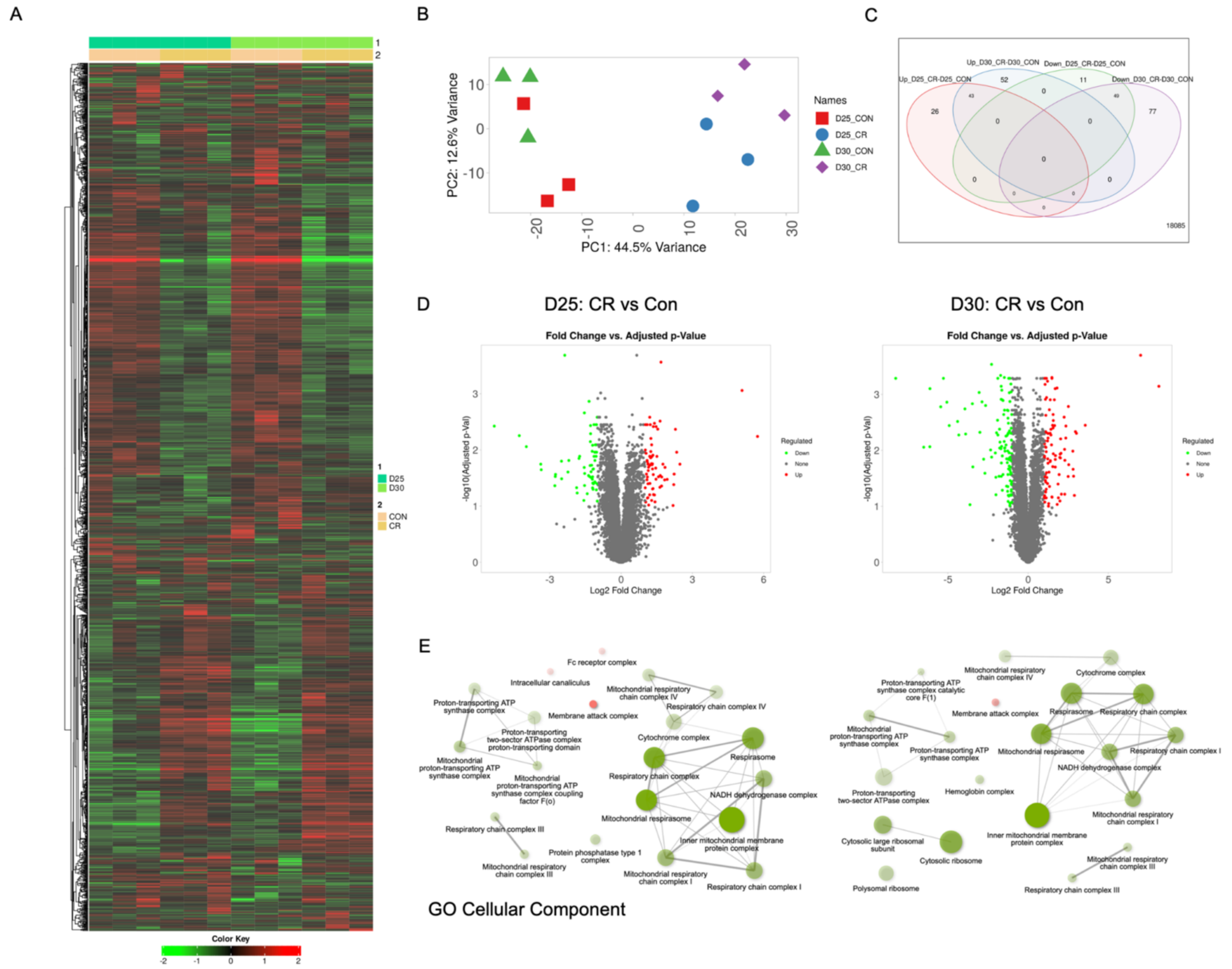


**Sup Fig 1. Gene regulation during CR. Hepatic mRNA levels by RNA-Seq are shown from mouse livers on days 25 and 30 of CR.** Over 18,000 genes were identified by RNA-Seq and analyzed by integrated Differential Expression & Pathway analysis (iDEP). **(A)** Heatmap represents the hierarchical clustering of the top 2000 genes by Pearson distance analysis. For comparison analysis, **(B)** PCA was carried out (PC1 is correlated with 2 (p=2.62e-06)) and **(C)** DEG1 (2-factor) generated Venn diagram (FDR cutoff: 0.1; Min fold-change: 2). **(D)** Volcano plot of associated DEGs. **(E)** Pathway analysis of associated DEGs by GO Cellular component analysis (Green is down in CR, Red is up in CR). Unlike protein FSR data, mRNA expression does not exhibit a marked shift in day 30 vs 25 livers between Con and CR groups.

A 10 Weeks Old

| | RER | HEAT | ZTOT | XTOT | XAMB |
|---|---|---|---|---|---|
| Con | 1.04 ± 0.017 | 0.27 ± 0.007 | 129.0 ± 43.0 | 495.6 ± 63.7 | 262.3 ± 41.1 |
| NO- | 1.01 ± 0.028 | 0.29 ± 0.012 | 109.0 ± 8.9 | 471.6 ± 51.3 | 238.2 ± 28.2 |
| CR | *0.95 ± 0.008 | *0.19 ± 0.010 | 222.9 ± 47.5 | 755.0 ± 69.5 | 453.9 ± 52.8 |
| NO-CR | *1.00 ± 0.013 | *0.19 ± 0.013 | 97.5 ± 31.5 | 429.3 ± 66.0 | 231.1 ± 44.2 |

B 22 Weeks Old

| | RER | HEAT | ZTOT | XTOT | XAMB |
|---|---|---|---|---|---|
| Con | 0.97 ± 0.025 | 0.43 ± 0.013 | 109.5 ± 38.1 | 476.0 ± 81.7 | 213.2 ± 55.2 |
| NO- | *0.91 ± 0.030 | *0.48 ± 0.012 | 97.9 ± 15.0 | 432.3 ± 73.6 | 184.3 ± 27.0 |
| CR | *0.91 ± 0.014 | *0.32 ± 0.012 | 76.8 ± 19.1 | 431.5 ± 56.9 | 200.8 ± 40.5 |
| NO-CR | *0.90 ± 0.010 | *0.33 ± 0.013 | 157.5 ± 61.4 | 644.4 ± 77.9 | 288.3 ± 58.0 |

*p<0.05, Student T-test

**Sup Fig 2. Table of Metabolic Cage Outcomes.** CLAMS assessment of mice. Metabolic assessment was performed in metabolic cages before 10 **(A)** or 22 **(B)** weeks of intervention. Metabolic Cages were used to assess the RER, HEAT, and movement scores of mice (12 total, n=4 per group). Animals were placed on a CLAMS system and their metrics were taken for 3 nights (dark) and 2 days (light). Dark and Light values were averaged together (*p<0.05 to Con).

| Timepoints: | D0 | D9 | D14 | D20 | D25 | D27 | D28 | D29 | D30 | D31 | D32 | D40 | D42 | D73 | D170 |
|---|---|---|---|---|---|---|---|---|---|---|---|---|---|---|---|
| Down in CR vs Con | 45.5% | 63.6% | 52.7% | 36.4% | 63.6% | 85.5% | 69.1% | 49.1% | 67.3% | 87.3% | 74.5% | 98.2% | 94.5% | 87.3% | 85.5% |
| Up in CR vs Con | 54.5% | 36.4% | 47.3% | 63.6% | 36.4% | 14.5% | 30.9% | 50.9% | 32.7% | 12.7% | 25.5% | 1.8% | 5.5% | 12.7% | 14.5% |

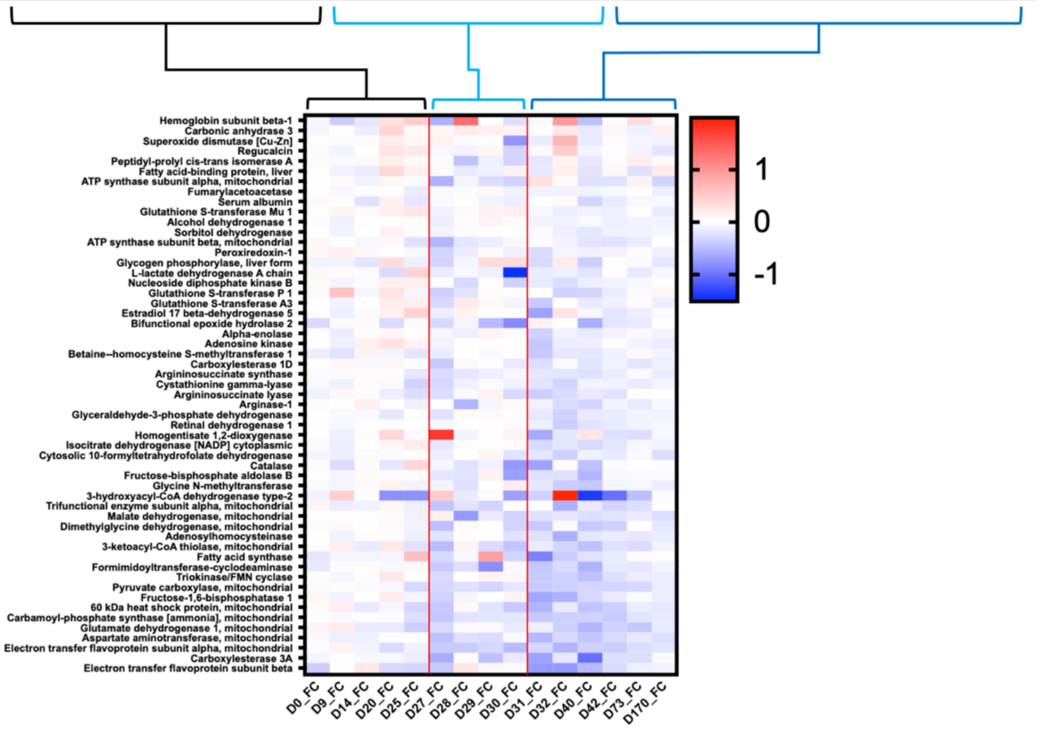


**Sup Fig 3. Fractional Synthesis Rates of Hepatic Proteins that are Present in All Time Points Across the Time Course.** Data show the fold change(log2FC) difference between CR and Con, with blue color being a decrease in FSR values under CR and red representing an increase in FSR under CR. Table represents the percentage of these proteins being up or downregulated during CR.

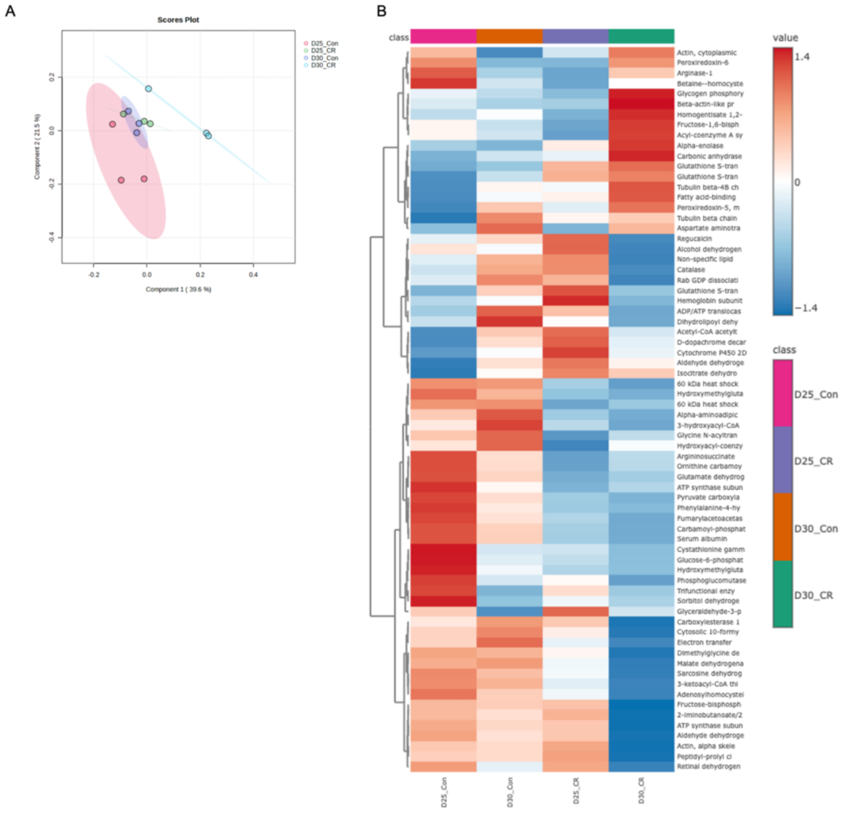


**Sup Fig 4. Label-Free Quantitative Proteomics and Calculation of WPAS Rates.** Peptides were aggregated into parent proteins based on the top 30% intensity, excluding single-peptide proteins. Protein-level variability and clustering were examined using a second Pearson cross-correlation and PCA, performed with InfernoRDN for Proteomics, to generate the normalized signal intensity quotient (Q). The Within Proteome Absolute Synthesis (WPAS) rate was derived by multiplying the fractional synthesis rate (f-value) by Q, reflecting the relative mass of newly synthesized protein within the proteome. The represented protein data was normalized by taking the WPASR values and performing a log2 transformation followed by mean-centering. Data was then subjected to Pearson cross-correlation analysis for quality control. **(A)** Principal Component Analysis (PCA, via partial least squares discriminant analysis) reveals distinct separation between the proteomic profiles of days 25 and 30 under caloric restriction. **(B)** Heatmap comparison between Day 25 Control, Day 30 Control, Day 25 CR, and Day 30 CR for aligned corresponding proteins, clustered using Ward's method with Euclidean distance, derived from the original data source.

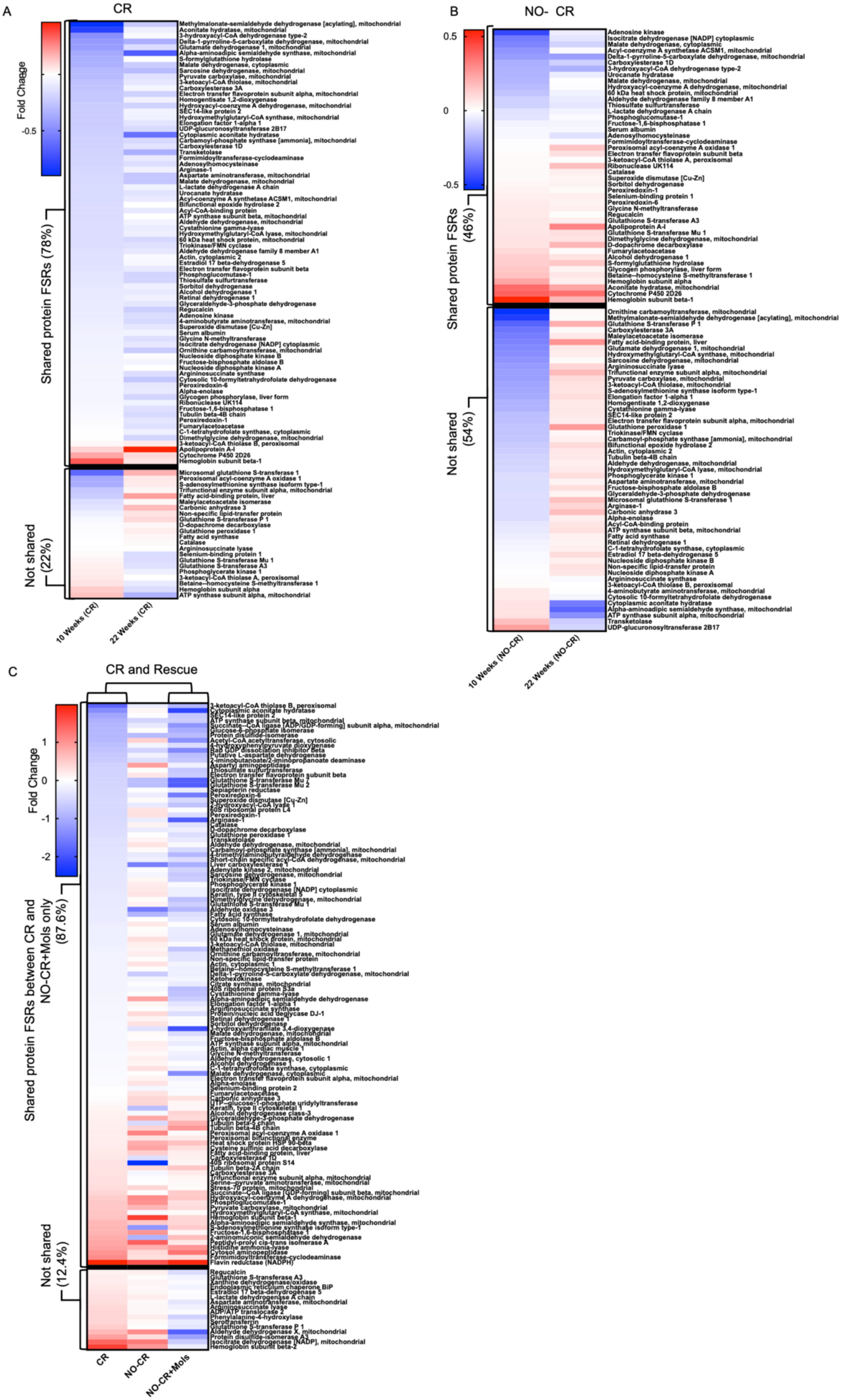
A
CR
Fold Chage
-0.5
Shared protein FSRs (78%)
Not shared (22%)
10 Weeks (CR)
22 Weeks (CR)
B
NO- CR
0.5
0
-0.5
Shared protein FSRs (46%)
Not shared (54%)
10 Weeks (NO-CR)
22 Weeks (NO-CR)
C
CR and Rescue
Fold Change
1
0
-1
-2
Shared protein FSRs between CR and NO-CR+Mols only (87.6%)
Not shared (12.4%)
CR
NO-CR
NO-CR+Mols

**Sup Fig 5. Comparison of Proteins Mediated by CR. (A)** Heatmap comparison of the fold-change differences shows the change in fractional synthesis rates (FSR) under CR compared to the control. Blue indicates a decrease in FSR in CR compared to the control, and red indicates an increase. At both 10 weeks and 22 weeks of CR, there is a high degree of consistency in the regulation of the hepatic proteome, with 78% of proteins being consistent between the two time points. **(B)** Heatmap comparison of NO- CR at 10 weeks and 22 weeks shows lack of consistent changes in FSR under CR without NO support, with 54% of proteins being inconsistent between the two timepoints. **(C)** Heatmap comparison of NO rescue under CR with Molsidomine (Mols) shows that only 12.4% of proteins are inconsistent between CR and NO-CR+Mols (rescue of CR).
